# A Comparative Analysis of Naïve Exosomes and Enhanced Exosomes with a Focus on the Treatment Potential in Ovarian Disorders

**DOI:** 10.3390/jpm14050482

**Published:** 2024-04-30

**Authors:** Mohammad Mousaei Ghasroldasht, Farzana Liakath Ali, Hang-Soo Park, Morteza Hadizadeh, Shao Huan Samuel Weng, Allen Huff, Somayeh Vafaei, Ayman Al-Hendy

**Affiliations:** 1 Department of Obstetrics and Gynecology, University of Chicago, Chicago, IL 60637, USA; mmghasroldasht@bsd.uchicago.edu (M.M.G.); farzanabegum.liakathali@bsd.uchicago.edu (F.L.A.); hspark06@scnu.ac.kr (H.-S.P.); somayehv@uchicago.edu (S.V.); 2 Department of Biomedical Science, Sunchon National University, Suncheon 57922, Republic of Korea; 3 Physiology Research Center, Institute of Neuropharmacology, Kerman University of Medical Sciences, Kerman 76198-13159, Iran; 4 Proteomics Platform, Office of Shared Research Facilities, Biological Sciences Division, University of Chicago, Chicago, IL 60637, USA; samuelweng@uchicago.edu (S.H.S.W.); allenh@uchicago.edu (A.H.)

**Keywords:** enhanced exosomes, ovarian cancer, polycystic ovary syndrome, poor ovarian reserve, premature ovarian insufficiency

## Abstract

Exosome-based therapy has emerged as a promising strategy for addressing diverse disorders, indicating the need for further exploration of the potential therapeutic effects of the exosome cargos. This study introduces “enhanced exosomes”, a novel type of exosomes developed through a novel cell culture system. These specific exosomes may become potent therapeutic agents for treating ovarian disorders. In this study, we conducted a comparative analysis of the protein and miRNA cargo compositions of enhanced exosomes and naïve exosomes. Our findings revealed distinct cargo compositions in enhanced exosomes, featuring upregulated proteins such as EFEMP1, HtrA1, PAM, and SDF4, suggesting their potential for treating ovarian disorders. MicroRNA profiling revealed that miR-1-3p, miR-103a-3p, miR-122-5p, miR-1271-5p, miR-133a-3p, miR-184, miR-203a-3p, and miR-206 are key players in regulating ovarian cancer and chemosensitivity by affecting cell cycle progression, cell proliferation, and cell development. We examined polycystic ovary syndrome and premature ovarian insufficiency and identified the altered expression of various miRNAs, such as miR-125b-5p and miR-130b-3p, for diagnostic insights. This study highlights the potential of enhanced exosomes as new therapeutic agents for women’s reproductive health, offering a detailed understanding of the impact of their cargo on ovarian disorders.

## 1. Introduction

Extracellular vesicles (EVs), complex structures that are released by cells into the extracellular space, are classified based on their size and biogenesis [1]. These vesicles encapsulate a cytosol devoid of organelles within a lipid bilayer membrane and are thus incapable of replication and coalescing into particles within the submicron range (30–5000 nm) [2]. The three main classes of EVs are (a) apoptotic bodies, ranging from 800 to 5000 nm in diameter; (b) microvesicles, also known as shedding MVs, which are large membranous vesicles with diameters ranging from 100 nm to 800 nm; and (c) exosomes, with diameters of 30–160 nm [2,3]. Initially discovered in the 1980s, exosomes were found to have an endosomal origin and are secreted by reticulocytes [4]. Notably, exosomes have a density of 1.13–1.19 g/mL, further distinguishing them as a specific type of EV [5].

Exosomes play a crucial role in intercellular communication and are actively released by various cells, tissues, and bodily fluids such as plasma, urine, saliva, tears, semen, and breast milk [6,7,8,9,10,11,12]. Functioning as molecular cargos, these small vesicles contain a diverse array of bioactive molecules, including cytoplasmic proteins, membrane proteins, cytokines, chemokines, cellular receptors, non-coding RNAs, mRNAs, and miRNAs [13,14]. The Vesiclepedia database has a comprehensive exosomal cargo list that encompasses 566,911 proteins, 27,692 mRNAs, 22,858 miRNAs, 3839 lipids, and 167 DNAs, emphasizing the extensive and dynamic nature of exosomal content (accessible online: http://microvesicles.org/, accessed on 10 February 2024). Facilitating the intricate exchange of information between cells, exosomes actively engage in the packaging and transportation of molecules. Operating as both paracrine and endocrine signaling agents, exosomes intricately govern cell-to-cell communications, affecting autocrine and paracrine cellular phenotypes [15].

In recent years, exosomes have been studied with a focus on three applications: as diagnostic markers, especially for the early detection and screening of cancers [14]; as drug delivery agents [16]; and as therapeutic tools [16]. Influenced by the cell of origin, exosomes contain diverse contents that undergo further variations under distinct physiological and pathological conditions [5]. By leveraging their nonimmunogenic properties and facilitating passage through biological and physical barriers, exosomes have emerged as promising candidates for delivering tailored molecules to treat specific disorders [17]. The strategic loading of ideal cargo within the lumen or on the surface increases the therapeutic potential of the cargo. Various methodologies, including incubation, transfection of donor cells, and physical treatments such as electroporation, sonication, extrusion, and the chimeric exosome method, have been employed in numerous studies to induce specific small molecules in the exosome lumen, aiming to increase the therapeutic efficacy for specific diseases [18]. However, inducing small molecules may increase the therapeutic efficacy of exosome-based therapy but has limitations. For instance, our knowledge of all small molecules affecting tissue regeneration is incomplete, and even if these small molecules are identified, loading all these small molecules simultaneously in exosomes using current cargo-loading strategies is challenging.

To address this challenge, for the first time, we have introduced a new technique to produce exosomes with a specialized cargo designed specifically for the repair of ovarian disorders. These exosomes, termed enhanced exosomes, are derived from umbilical cord stem cells under unique conditions. In this study, we compared the miRNA and protein profiles of umbilical cord stem cell-derived exosomes (naïve exosomes) and enhanced exosomes, with a focus on the treatment potential in ovarian disorders. This approach aims to provide a targeted and efficient strategy for therapeutic intervention in ovarian disorders, highlighting the potential of exosomes as precision medical tools in the field of regenerative medicine.

## 2. Materials and Methods

### 2.1. Cell Culture and Exosome Purification

We utilized mesenchymal stem cells derived from the human umbilical cord (hUC-MSCs) that were obtained from RoosterBio (Frederick, MD, USA). These cells were specifically isolated from the perivascular Wharton’s jelly region of the human umbilical cord. The cultivation and expansion of these cells adhered to the guidelines provided by RoosterBio and were conducted in RoosterNourish-MSC-XF medium (RoosterBio, Frederick, MD, USA) until the cells reached 80% confluence. After reaching the third passage at 80% confluence, the hUC-MSCs were washed three times with phosphate-buffered saline (PBS) and subsequently cultured with RoosterCollect-EV Pro™ medium (RoosterBio, Frederick, MD, USA) for 48 h. The resultant culture medium, enriched with secreted exosomes, underwent a series of centrifugation steps: 500× *g* for 5 min at 4 °C to eliminate cell debris, followed by 2000× *g* for 20 min to remove apoptotic bodies, and 10,000× *g* for 30 min to exclude microvesicles. The purification of exosomes was performed with the poly-ethyl glycol (PEG)-based precipitation method (ExoQuick-TC, System Biosciences, Palo Alto, CA, USA), which strictly adhered to the manufacturer’s protocol. Briefly, 1 mL of ExoQuick-TC was added to 5 mL of culture media, and the mixture was incubated overnight at 4 °C. The ensuing centrifugation at 1500× *g* for 30 min resulted in the pelleting of exosomes. The resulting pellet was resuspended in 200 µL of PBS, and the quality and quantity of the exosomes were evaluated using NanoTracking Particle analysis with a NanoSight NS300 system (Malvern Panalytical, United Kingdom). The purified exosomes were stored at −80 °C and subjected to no more than one freeze–thaw cycle.

The immortalized human granulosa cell line (HGrC1), a generous gift from Dr. A. Iwase (Nagoya University, Nagoya, Japan), was cultured in DMEM-F12 supplemented with 10% fetal bovine serum, and 1% penicillin-streptomycin (Thermo Fisher Scientific Inc., Waltham, MA, USA).

To produce enhanced exosomes, we cultured hUC-MSCs with HGrC1 cells in accordance with a system protected by intellectual property rights (patent number: PCT/US2022/073467). The culture medium enriched with exosomes was harvested and subjected to a series of sequential centrifugation steps: centrifugation at 500× *g* for 5 min, 2000× *g* for 20 min, and 10,000× *g* for 30 min to eliminate debris, apoptotic bodies, and microvesicles, respectively. Exosome purification was carried out using ExoQuick-TC, strictly following the manufacturer’s instructions. The quality and quantity of the exosomes were thoroughly analyzed using a NanoSight NS300 system.

To validate the distinctive morphology of both naïve exosomes and enhanced exosomes, we performed transmission electron microscopy (TEM) studies. In brief, exosomes were applied to a copper mesh grid coated with a carbon film for 1 min and subsequently negatively stained with 0.75% uranyl formate for 45 s. After the drying process, TEM imaging was conducted using an FEI Tecnai G2 F30 system operating at 300 kV. Western blot analysis, based on the manufacturer’s protocol, was used to validate the presence of exosomal markers, including CD9, CD63, and CD81 (System Bioscience, Palo Alto, CA, USA).

### 2.2. Proteomics

#### 2.2.1. Chemicals and Reagents

Tris(2-carboxyethyl)phosphine (TCEP; C4706), iodoacetamide (IAA; I6125), and calcium chloride (C5080) were obtained from Sigma. Ammonium bicarbonate (BP2413), LC-MS grade acetonitrile (A955-4), and water (W6-4) were obtained from Fisher Scientific. Formic acid (cat. no. 94318) was sourced from Honeywell. Trypsin/LysC (sequencing grade; Promega; V5073) was obtained.

#### 2.2.2. S-Trap Digestion Protocol

Protein extraction was conducted from 40 billion particles of both naïve exosomes and ovarian-specific exosomes. For this procedure, exosome samples in PBS were denatured using equal volumes of 2× S-Trap lysis buffer (10% SDS, 100 mM TEAB, pH adjusted to 7.55 using 12% phosphoric acid). The samples were subjected to bead beating and reduction with 20 mM TCEP (200 mM TCEP in 770 mM TEAB, pH 7.8) at 65 °C for 30 min. Subsequently, the reduced proteins were alkylated with 80 mM iodoacetamide in the dark for 30 min at room temperature. Acidification of the samples was performed using 12% phosphoric acid (final concentration 1.2%), followed by dilution with a 6× volume of S-Trap binding buffer (methanol containing 100 mM TEAB, pH adjusted to 7.2 using 12% phosphoric acid). The diluted sample was loaded onto an S-Trap micro column using a microcentrifuge with a flow-through waste collector. Finally, SDS was completely eliminated from the sample by washing the filter three times with 160 μL of S-Trap binding buffer. The purified protein samples on the filter were then subjected to digestion using 2 μg of trypsin/Lys-C (#V5073, Promega, Madison, WI, USA) in 50 mM ammonium bicarbonate and 0.5 mM CaCl2 at 37 °C overnight. The digested peptides were collected by washing the filter in three steps with 40 μL of 50 mM ammonium bicarbonate, 40 μL of 0.15% formic acid, and 40 μL of 0.15% formic acid in 60% acetonitrile. The eluate was dried in vacuo and stored at −80 °C prior to analysis.

#### 2.2.3. LC-MS Parameters

The samples were analyzed with an Exploris 480 mass spectrometer along with an UltiMate 3000 liquid chromatography system (Thermo Fisher Scientific). A MonoCap column from GL Sciences, measuring 50 cm in length and 0.75 mm in inner diameter (Cat. No. 5020-10006), was used, with a flow rate of 500 nL/min and a constant temperature of 25 °C.

For the analysis, mobile phase A (0.15% formic acid in water) and mobile phase B (0.15% formic acid in 100% acetonitrile) were used. The gradient spanned 130 min: 5% B for 5 min, transitioning from 5% to 22% B over 100 min, followed by a shift from 22% to 34% B over 19 min, and a rapid transition from 34% to 95% B in 1 min. The mixture was held at 95% B for 5 min.

A full-scan MS spectrum ranging from 350 to 1650 *m*/*z* was collected at a resolution of 60,000 at *m*/*z* 200. The maximum injection time was set to 50 ms, with an AGC target value of 3e6. The cycle time for data acquisition was set to 3 s, and the intensity threshold was set at 5e4.

For MS/MS scans, a resolution of 15,000 was utilized, with the maximum acquisition time set to auto and an AGC target value of 4e4. The isolation window at the Orbitrap cell was set to 1.6 *m*/*z*, and the first mass was set to 110 *m*/*z*. The collision energy for HCD was set to 32. A dynamic exclusion duration of 30 s was implemented. Charge states 2 to 7 were included, while unassigned charge states were ignored. The heated capillary temperature was set to 300 °C.

#### 2.2.4. Proteomics Analysis

Raw MS data were processed and searched using Proteome Discoverer (version 3.0.0.757; Thermo Fisher Scientific) with the Sequest HT search engine. A precursor mass tolerance of 10 ppm and fragment mass tolerance of 0.02 Da were applied. The human database was downloaded from UniProt.

A 1% FDR cutoff, estimated by Percolator, was applied to filter the data. Trypsin (full) was used as the enzyme, with a maximum of 3 mass cleavages, and peptide lengths ranging from 4 to 45. Carbamidomethyl (+57.021 Da on C) was selected as a fixed modification, while oxidation (+15.995 Da on M), deamidated (+0.984 Da on N and Q), protein N-terminal Met-loss (−131.040 Da), acetyl (+42.011 Da on N-terminus), and protein N-terminal Met-loss+acetyl (−89.030 Da) were considered dynamic modifications.

Gene set enrichment analysis (GSEA) provides crucial insights into the biological functionality of organisms, focusing on Gene Ontology (GO) and biological pathway analyses. The GOplot and ClusterProfiler packages in R software (V 4.4.0) allow a thorough examination of the final list, facilitating a comprehensive investigation of both GO and pathway enrichment. The analysis was performed with a significance threshold of *p* < 0.05, ensuring that only the most statistically significant outcomes were considered.

### 2.3. MiRNA Sequencing

RNA was extracted from 40 billion particles of both naïve exosomes and enhanced exosomes using the SeraMirTM Kit (System Biosciences, Palo Alto, CA, USA) following the manufacturer’s protocol. Following a rigorous quality control assessment of the isolated RNA, a small RNA sequencing assay was carried out by Novogene Company (San Diego, CA, USA) in accordance with the established protocol (Novogene mRNA-seq Services).

In essence, 3′ and 5′ adaptors were ligated to the respective ends of small RNAs, and first-strand cDNA was synthesized after hybridization with the reverse transcription primers. The resulting double-stranded cDNA library was generated through PCR enrichment. After purification and size selection, libraries with insertions ranging from 18 to 40 bp were prepared for sequencing on Illumina platforms with PE50.

Quantification and size distribution analysis of the libraries were performed using Qubit, real-time PCR, and bioanalyzer methodologies. Subsequently, the quantified libraries were pooled and subjected to sequencing on an Illumina platform, determined by the effective library concentration and the requisite data volume. The bioinformatics team at Novogene conducted a thorough analysis involving the mapping and identification of known miRNAs using Bowtie (version 1.0.1) and mirDeep2, respectively. Novel miRNAs were predicted through miREvo (version MirEvo_v1.1) and mirDeep2. For animal target prediction, miRanda (version miRanda-3.3a) and RNAhybrid (version RNAhybrid v2.0) were used. Additionally, GO and KEGG enrichment analyses were carried out using clusterProfiler (version 3.8.1) with an adjusted *p* value <0.05.

## 3. Results

### 3.1. Exosome Characteristics

**Figure 1:**
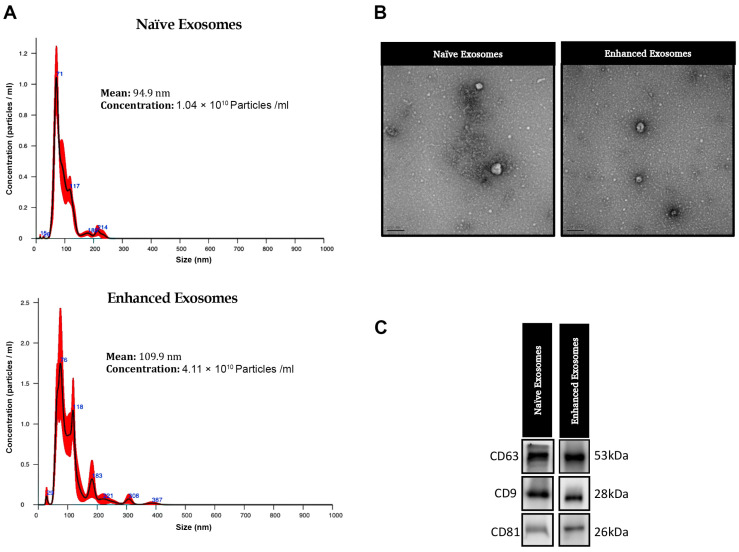
Characterization of exosomes derived from umbilical cord mesenchymal stem cells under conventional conditions (naïve exosomes) and specific cell culture conditions (enhanced exosomes). (**A**) Nanoparticle tracking analysis (NTA), revealing the average size and number of particles. (**B**) Transmission electron microscopy (TEM) images illustrating the morphology of naïve and enhanced exosomes. (**C**) Western blot (WB) analysis of exosomal markers (CD63, CD9, and CD81) in naïve and enhanced exosomes.

Extracellular vesicles in their original state were obtained from human umbilical cord mesenchymal stem cells (hUC-MSCs) cultured under conventional conditions, while enhanced exosomes were harvested from hUC-MSCs exposed to unique conditions. A nanoparticle tracking analysis (NTA) revealed that the particle size ranged from 30 to 200 nm, with an average size of 109.9 nm for naïve exosomes and 94.9 nm for enhanced exosomes (Figure 1). Notably, the concentration of enhanced exosomes was greater than that of naïve exosomes for the same number of seeded cells, indicating that the cells cultured in our new cell culture system secreted more particles (1.04 × 10^10^ particles/mL naïve exosomes versus 4.11 × 10^10^ particles/mL enhanced exosomes). The morphology of the isolated exosomes was evaluated using TEM, which revealed no discernible differences in shape between the naïve and enhanced exosomes. The Western blotting analysis confirmed the expression of exosomal marker proteins, including CD9, CD63, and CD81 (System Bioscience, USA), in both naïve and enhanced exosomes.

### 3.2. Bioinformatics Analyses of Proteins Derived from Naïve Exosomes and Enhanced Exosomes

**Figure 2:**
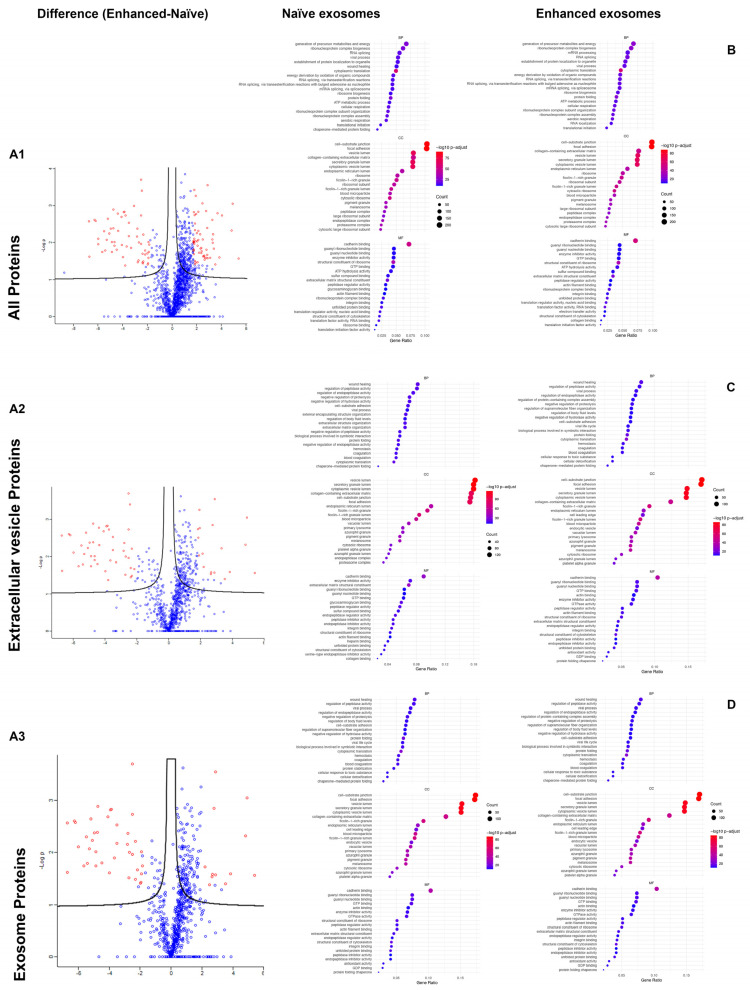
Protein analysis of naïve exosomes and enhanced exosomes. (**A1**–**A3**) Volcano plots of log2 fold changes (*x*-axis) and their associated −log10 transformed *p*-values (*y*-axis) for all identified proteins, extracellular vesicle proteins, and exosome proteins (Red dots). (**B**–**D**) GO analysis of all the identified proteins, extracellular vesicle proteins, and exosome proteins in the naïve and enhanced exosome groups.

A comparison between naïve exosomes and enhanced exosomes is presented in Figure 2A; a volcano plot was used to show the variations in protein expression between each pair of groups. The proteins were categorized into three groups: all proteins, extracellular vesicle-specific proteins, and exosome-specific proteins. A chord plot and cnetplot were generated to identify enriched genes and pathways according to the Gene Ontology (GO) and KEGG enrichment analyses, with an adjusted *p* value < 0.05. Like enhanced exosomes, naïve exosomes contained proteins involved in the generation of precursor metabolites and energy and ribonucleoprotein complex biogenesis in the biological process category (Figure 2B). For cellular components, both naïve and enhanced exosomes were associated with cell–substrate junctions and focal adhesions. In terms of molecular function, cadherin binding was the primary function for both types of exosomes in all protein categories (Figure 2B).

In the extracellular vesicle protein category, both naïve and enhanced exosomes played roles in wound healing and the regulation of peptide activity in biological processes. Cadherin binding was a significant molecular function for both groups. However, in cellular components, naïve exosomes had more proteins involved in vesicle lumen and secretory granule lumen, while enhanced exosomes had more proteins involved in cell–substrate junction and focal adhesion (Figure 2C). In the exosome category, the proteins associated with biological processes, cellular components, and molecular functions were consistent between the naïve and enhanced exosome groups (Figure 2D).

**Figure 3:**
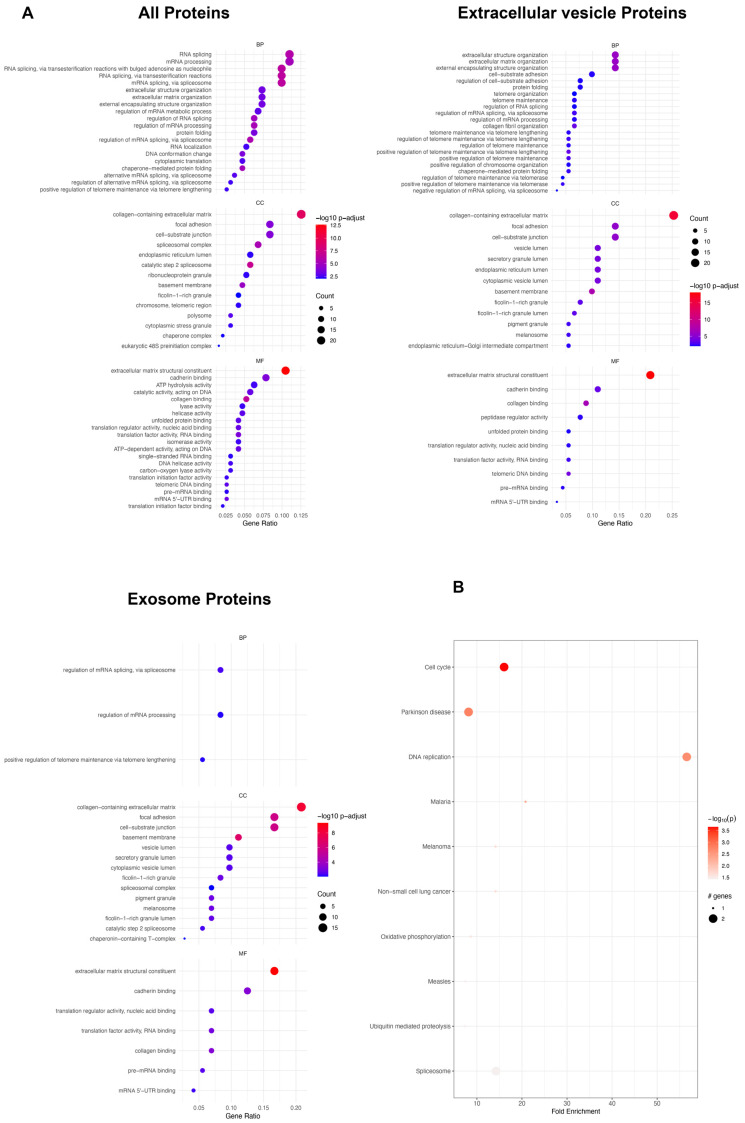
Protein analysis of proteins significantly overexpressed in enhanced exosomes. (**A**) GO analysis of all the identified proteins, extracellular vesicle proteins, and exosome proteins. (**B**) KEGG pathway.

To further elucidate the differences between enhanced and naïve exosomes, we compared the expression of proteins significantly overexpressed in the enhanced exosomes. There were 191 proteins significantly overexpressed in all protein categories, 91 in the extracellular vesicle category, and 72 in the exosome category (Supplementary Table S1). According to the analysis of all protein categories, the GO analysis revealed involvement in biological processes such as RNA splicing and mRNA processing (Figure 3A). In terms of cellular components, there was a greater representation of proteins associated with the collagen-containing extracellular matrix, while the molecular functions were predominantly related to the extracellular matrix structural constituent (Figure 3A). In the extracellular vesicle category, proteins were linked to biological processes involving extracellular structure organization, extracellular matrix organization, and external encapsulating structure organization. The collagen-containing extracellular matrix dominated the cellular component, and the molecular function was primarily associated with extracellular matrix structural constituents (Figure 3A). Within the exosome category, proteins shared similarities with those in the protein and extracellular vesicle categories in terms of cellular components and molecular functions. However, proteins in this category play specific roles in the regulation of mRNA splicing and mRNA processing within biological processes (Figure 3A). The KEGG pathway analysis revealed predominant enrichment in the cell cycle for all proteins in the enhanced exosome group (Figure 3A), with additional pathways such as the PI3K-Akt signaling, AMPK signaling, apoptosis, RNA transport, cGMP-PKG signaling, mTOR signaling, and DNA replication pathways being significantly overexpressed in the enhanced exosome group compared to the naïve exosome group (Supplementary Table S2).

### 3.3. Bioinformatics Analyses of miRNAs Derived from Naïve Exosomes and Enhanced Exosomes

**Figure 4:**
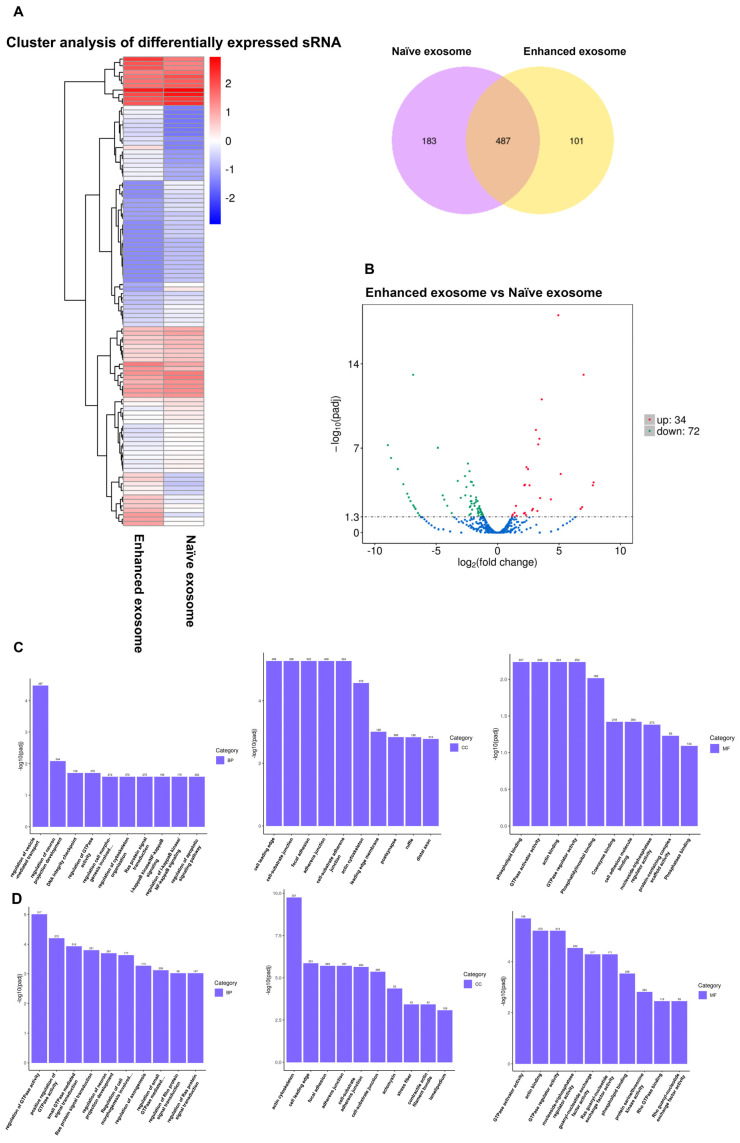

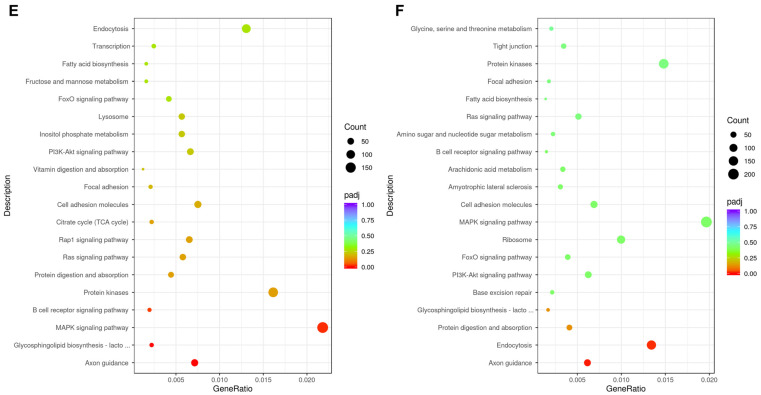
MiRNA analysis of naïve exosomes and enhanced exosomes. (**A**) Heatmap and Venn diagrams of the miRNA levels in naïve and enhanced exosomes: 487 common miRNAs, 183 miRNAs unique to naïve exosomes, and 101 miRNAs unique to enhanced exosomes. (**B**) Volcano plots of log2 fold changes (*x*-axis) and their associated -log10 transformed *p*-values (*y*-axis) for all identified miRNAs. (**C**) GO analysis of all the detected miRNAs. (**D**) GO analysis of miRNAs significantly upregulated in the enhanced exosomes. (**E**) KEGG analysis of all the detected miRNAs. (**F**) KEGG analysis of miRNAs significantly upregulated in the enhanced exosomes.

A Venn diagram describing the miRNA profile of naïve exosomes versus enhanced exosomes indicated the presence of 487 common miRNAs, 183 miRNAs unique to naïve exosomes, and 101 miRNAs unique to enhanced exosomes (Figure 4A). To assess the notable differences between the two exosomal miRNAs, we generated a volcano plot to identify the uniquely expressed or overexpressed miRNAs in each group (Figure 4B). The data revealed that 34 miRNAs exhibited significant upregulation in the enhanced exosomes compared to the naïve exosomes, while 74 miRNAs displayed significant downregulation in the enhanced exosomes in comparison to the naïve exosomes (fold change > 2, and *p*-value < 0.05, see Supplementary Table S3 for a complete list). Given that each miRNA can target several genes, influencing their expression or translation, these diverse miRNAs are likely to play roles in various crucial mechanisms related to ovarian regeneration. Supplementary Table S4 provides an overview of the target genes of a selection of well-known miRNAs that are overexpressed in enhanced exosomes compared to naïve exosomes (the network of miRNAs and target genes is illustrated in Supplementary Figure S2).

Subsequently, a comprehensive analysis of the biological processes, cellular components, and molecular functions of all the miRNAs was conducted through Gene Ontology (Figure 4C). The data indicated that the majority of the miRNAs were involved in the regulation of vesicle-mediated transport in the biological process category. In terms of cellular components, most miRNAs were implicated in cell leading edge, cell–substrate junction, focal adhesion, and cell–substrate adherent junction processes. Concerning molecular function, the predominant roles of miRNAs were related to phospholipid binding, GTPase activator activity, actin binding, and GTPase regulator activity.

A further GO analysis of the miRNAs that exhibited significant upregulation in the enhanced exosomes compared to the naïve exosomes revealed enriched biological processes, with the identified miRNAs being predominantly associated with the regulation of GTPase activity. Among the cellular component terms, the majority of the identified miRNAs were involved in the actin cytoskeleton, while in terms of molecular function, GTPase activator activity was the most common category (Figure 4D).

We subsequently performed a KEGG analysis, and the results revealed that miRNAs expressed in both naïve and enhanced exosomes play roles in axon guidance, endocytosis, protein digestion and absorption, and glycosphingolipid biosynthesis (Figure 4E). Specifically, among the upregulated miRNAs in the enhanced exosomes, a significant proportion were associated with axon guidance, glycosphingolipid biosynthesis, the MAPK signaling pathway, and the B-cell receptor signaling pathway (Figure 4F).

## 4. Discussion

A woman’s overall well-being is intricately linked to the health of her ovaries, which are affected by range of conditions, such as cancer, cysts, polycystic ovary syndrome (PCOS), poor ovarian reserve (POR), and premature ovarian insufficiency (POI). These multifaceted conditions are influenced by a combination of genetic and environmental factors [19]. The molecular intricacies governing processes within the ovary, including estrogen synthesis, oocyte maturation, and cumulus functions, are critical for maintaining reproductive health. Recently, exosome-based products have emerged as promising therapeutic agents for various disorders, including ovarian conditions [20]. This study delves into the roles of cargo molecules within exosomes, both in the lumen and on the surface, providing valuable insights into the molecular mechanisms underlying the treatment of ovarian dysfunction. In this study, we produced a new type of exosome termed “enhanced exosomes” using a novel approach involving specific cell culture conditions. The objective of this study was to determine the unique composition of enhanced exosomes compared to that of naïve exosomes, providing a comprehensive profile of miRNAs and proteins.

Through meticulous analysis and the use of tools such as volcano plots, we found the significant overexpression of specific proteins and miRNAs in the enhanced exosomes. These findings suggested the potential involvement of these genes in the treatment of ovarian disorders. According to an extensive literature review, specific proteins and miRNAs recognized for their overexpression in these exosomes have demonstrated therapeutic potential across various ovarian disorders. The expression of several proteins, including EFEMP1 (EGF-containing fibulin extracellular matrix protein 1), HtrA1 (high temperature requirement A), PAM (Peptidyl-glycine alpha-amidating monooxygenase), and SDF4 (stromal cell-derived factor 4), were significantly greater in enhanced exosomes than in the naïve exosomes. Previous studies have confirmed the important role of these compounds in treating ovarian disorders through different pathways.

EFEMP1, an extracellular matrix protein, is crucial for maintaining the structural integrity of the extracellular matrix. While EFEMP1 has been associated with various carcinomas, its role in ovarian cancer is unclear [21]. Previous studies have indicated that the activation of the AKT signaling pathway can aid in treating POI [22]. Compared with that in naïve exosomes, the expression of EFEMP1 in enhanced exosomes was significantly greater, suggesting a potential impact on ovarian tissue functions.

HtrA1, a gene encoding a serine protease that plays a role in apoptosis and tissue remodeling, is implicated in various cellular processes. The dysregulation of HtrA1 may disrupt ovarian tissue homeostasis, potentially contributing to ovarian disorders. The reduced nuclear expression of HtrA1 is linked to a more favorable prognosis in patients with high-grade serous ovarian carcinoma [23]. Additionally, the degradation of the X-linked inhibitor of apoptosis protein (XIAP) by HtrA1 plays a role in the cellular response to chemotherapy, suggesting that restoring HtrA1 expression could be a promising therapeutic approach for treating ovarian cancer [24]. Based on the protein profiling data, the expression of HTRA1 was significantly greater in the enhanced exosomes than in the naïve exosomes, suggesting the potential increase in the therapeutic efficacy of enhanced exosomes for treating ovarian disorders.

Although the direct involvement of PAM in ovarian disorders has not been fully defined, it is a crucial protein known to play a significant role in ovulation and the response to estradiol. PAM is associated with the rise in progesterone levels during ovulation. Poulsen’s study reported an overexpression of PAM, peaking at 17–32 h, which corresponds with progesterone production in granulosa cells [25]. Our proteomics data revealed a significantly greater abundance of PAM in enhanced exosomes compared to naïve exosomes. This notable increase in PAM expression in enhanced exosomes suggests its potential role in treating patients with POI.

SDF4, which is responsible for encoding a stromal cell-derived factor, plays a role in protein folding and secretion. Given the critical importance of proper protein folding for the functionality of ovarian proteins, any dysregulation of SDF4 may contribute to ovarian dysfunction [26]. SDF4 exhibits a notable increase in expression in enhanced exosomes comprised to naïve exosomes, indicating a potential effect on the functions of ovarian tissue.

Among the myriad of regulatory molecules, microRNAs (miRNAs) are key modulators of gene expression. In the field of ovarian disorders, the encapsulation of miRNAs in exosomes is a promising strategy for therapeutic intervention. Through miRNA profiling, we observed the significant overexpression of several miRNAs in the enhanced exosomes compared to the naïve exosomes. Based on an extensive literature review, we have demonstrated that these overexpressed miRNAs have therapeutic potential for various ovarian disorders.

MiR-1-3p, a microRNA (miRNA), plays a nuanced role in ovarian function and disease, demonstrating both tumor-suppressive and oncogenic properties, contingent on the specific context. As a tumor suppressor, miR-1-3p obstructs cell proliferation, migration, and invasion, restricting the expansion of ovarian cancer cells by targeting crucial genes involved in cell cycle progression, such as c-Met and FZD7 [27,28]. Qu et al. demonstrated lower expression levels of miR-1 in ovarian cancer cell lines compared to non-neoplastic tissues. Additionally, they found that the overexpression of miR-1 in control cell lines led to a significant decrease in c-Met expression, a direct target of miRNA. This reduction in c-Met expression subsequently affected the expression levels of other factors, including p-Akt, p-ERK1/2, CDK4, and p-Rb, ultimately resulting in the inhibition of cell proliferation and arrest in the G1 cell cycle [27]. Zhang demonstrated that miR-1-3p binds to the 3′UTR site of FZD7 and reduces FZD7 expression in ovarian cancer cell lines. This reduction in FZD7 expression resulted in decreased cell viability and proliferation [28]. Previous studies have indicated the role of miR-1-3p as a tumor suppressor in different cancers through different mechanisms, such as upregulating SFRP1, repressing E2F5 and PFTAIRE protein kinase 1, and modulating BDNF and TrkB [29,30,31,32]. Moreover, miR-1-3p increases chemotherapy sensitivity in ovarian cancer cells by targeting genes associated with drug resistance [28]. Notably, Fu et al. observed the significant downregulation of miR-1-3p in ovarian cancer tissues compared to normal tissues [33]. The substantial overexpression of miR-1-3p in enhanced exosomes imparts a tumor-suppressive effect, contributing to their heightened therapeutic potential. Additionally, the presence of this miRNA enhances the sensitivity of ovarian cancer cells treated with enhanced exosomes to chemotherapy, a crucial aspect of the treatment for ovarian cancer.

MiR-103a-3p significantly influences ovarian cancer dynamics by suppressing CHI3L1, effectively inhibiting proliferation and angiogenesis. However, there is currently no established mechanism regarding the targeting of CHI3L1 by miR-103a-3p. Nevertheless, Lifen et al. demonstrated that CHI3L1 expression was downregulated in the group of cells with induced miR-103a-3p expression. This change strongly inhibits ovarian lymphatic metastasis and distant metastasis, potentially involving the TGF-β pathway [34]. The overexpression of miR-103a-3p in enhanced exosomes exerts a regulatory effect on controlling ovarian cancer.

MiR-122-5p is recognized for its tumor-suppressive functions in various cancers [35,36,37], and miR-125b-5p, known for its low expression in diverse tumor tissues [38], plays important roles in ovarian cancer. Previous studies have demonstrated that miR-122-5p inhibits the migration, invasion, and metastasis of ovarian cancer cells by targeting the P4HA1 gene [35]. Collagen, as a key component of the extracellular matrix, plays a crucial role in cancer progression and metastasis. P4HA1, a component involved in collagen maturation, has been implicated in these processes. Previous studies have indicated a significant decrease in P4HA1 levels in cells treated with miR-122b-5b [35]. Additionally, Chen et al. reported the downregulation of miR-125b-5p in ovarian cancer cells, emphasizing its role in inhibiting migration and invasion [39]. While the direct role of CD147 in ovarian cancer has not been fully elucidated, it is known to play a tumor-promoting role, facilitating tumor cell proliferation and invasion. Importantly, miR-125b-5p negatively regulates CD147 expression, thereby contributing to the inhibition of ovarian cancer cell migration and invasion [39]. Our miRNA sequencing analysis of the enhanced exosomes and naïve exosomes revealed a significantly greater expression of miR-122-5p in the enhanced exosomes. Given the confirmed role of miR-122-5p as a tumor suppressor that can control the migration and invasion of ovarian cancer cells, enhanced exosome secretion may be an effective strategy for the treatment of ovarian cancer.

MiR-1271-5p functions as a cancer suppressor, as demonstrated by Liu et al., and its overexpression is associated with the inhibition of ovarian cancer growth through the targeting of CCNG1 [40]. CCNG1 (cyclin G1) plays a crucial role in ovarian cancer progression, and miR-1271-5p targets the 3′UTR of CCNG1, leading to its inhibition. Moreover, this miRNA directly targets E2F5, a key player in DNA synthesis, negatively regulating the mTOR signaling pathway and hindering the development of ovarian cancer cells [41]. Additionally, miR-1271-5p directly targets TIAM1, a gene associated with metastasis, leading to the deactivation of the Notch signaling pathway and the consequent inhibition of ovarian cancer progression [41]. MiR-1271-5p was identified as another miRNA that is significantly overexpressed in enhanced exosomes compared to naïve exosomes, as indicated by our miRNA profiling results. Given the cancer suppressor effect and its inhibitory role in cancer progression associated with this miRNA, the enhanced exosome secretion, which is associated with the elevated expression of miR-1271-5p, holds therapeutic potential for the treatment of ovarian cancer.

A previous study indicated that miR-133a-3p expression is downregulated in ovarian cancer tissues compared with nontumor tissues. MiR-133a-3p negatively regulates the expression of PYGB by binding to the 3′-UTR of PYGB and ultimately inhibiting ovarian cancer development via the Wnt/B-catenin signaling pathway [42]. In a different study, miR-133a-3p was shown to act as a tumor suppressor and to inhibit the proliferation, differentiation, and motility of cancer cells via different mechanisms, such as targeting several oncogenes, targeting the EGFR/Akt signaling pathway, and inhibiting IGF1R expression [43,44,45]. MiR-133a-3p binds to the 3′UTR of EGFR, resulting in the decrease of EGFR protein expression levels. The reduced level of EGFR has an impact on suppressing the phosphorylation of AKT. Lower levels of phospho-AKT are associated with the induction of apoptosis in cancer cells [44]. MiR-133a plays diverse roles in controlling ovarian cancer through its involvement in various signaling pathways. Moreover, the overexpression of this miRNA in enhanced exosomes, compared to that in naïve exosomes, suggests the potential to increase the therapeutic efficacy of enhanced exosomes in the context of ovarian cancer.

MiR-184 is downregulated in ovarian cancer cells and tissues, and its overexpression induces apoptosis and inhibits cell proliferation [46,47]. The expression of miR-203a-3p is lower in ovarian cancer tissue than in normal tissue. Liu et al. demonstrated that miR-203a-3p is an anti-oncogenic factor in ovarian cancer. The overexpression of this miRNA in ovarian cancer cells inhibits cell proliferation, invasion, and migration; induces apoptosis; and arrests the cell cycle in the G1 phase by targeting ATM. This regulatory mechanism influences the Akt/GSK-3B/Snail signaling pathway, contributing to the control of ovarian cancer proliferation [48]. In a separate study, miR-203a-3p was reported to target CXCL1 at the mRNA level, leading to the inhibition of proliferation, migration/invasion, and angiogenesis in ovarian cancer cells [49]. This finding is attributed to the role of CXCL1, which, when attached to the cell surface receptor CXCR2, activates various pathways, including the PI3K/Akt, PLC/PKC, Ras/Erk, and JAK2/STAT3 signaling pathways [50]. MiR-206 plays a pivotal role in inhibiting ovarian cancer proliferation through diverse mechanisms, as confirmed in several studies. This molecule can directly target c-Met, suppressing the activation of the downstream AKT/mTOR signaling pathway [51]. The overexpression of KIF2A is positively correlated with biomarkers for cell invasion and migration, as well as longer overall survival time. However, miR-206 targets KIF2A, resulting in the inhibition of cancer cell proliferation, migration, invasion, and induction of apoptosis [52]. Additionally, miR-206 targets CCND1 and CCND2, inhibiting the proliferation, progression, migration, and invasion of ovarian cancer cells [53]. In estrogen-dependent tumors, this miRNA targets PFKFB3, regulating cancer cell proliferation through GLUT1, PFKFB3, and FAK [54]. MiR-184, miR-203a-3p, and miR-206, which are downregulated in ovarian cancer, play pivotal roles in controlling cancer cell proliferation, reducing metastasis, and inhibiting invasion through the targeting of various genes and signaling pathways. Notably, the significant overexpression of all three miRNAs in enhanced exosomes suggested a promising therapeutic strategy for ovarian cancer.

With respect to polycystic ovarian syndrome (PCOS), various studies have reported a notable reduction in the expression of miR-103a-3p, miR-106a-5p, and miR-125b-5p in PCOS patients compared to control subjects [55,56,57,58]. Changes in the expression of these genes were found to modulate pathways associated with axon guidance, MAPK signaling, endocytosis, circadian rhythms, and cancer. In PCOS, elevated Pak3 expression inhibits ERK1/2 activation, leading to reduced estradiol production, granulosa cell death, and increased testosterone production. MiR-125b-5p binds to Pak3, reducing its expression. Consequently, activated ERK1/2 induces estradiol production and inhibits granulosa cell death and testosterone production [58].

McAllister et al. conducted a target prediction analysis and found that miR-130b-3p may target PCOS-related genes, including DENND1, ZNF217, RAB5B, LHCGR, ERBB3, and KCNA4 [59]. The study indicated that DENND1A.V2 increased the expression of both CYP17A1 and CYP11A1, which are responsible for androgen production in the theca cells of PCOS patients. MiR-130b-3p was implicated in the translational regulation and expression of DENND1A.V2. Pathway and network analyses suggested that miR-130b-3p inhibits the upregulation of DENND1A and RAB5B, influencing the expression of theca cell LH receptors at the cell surface. Additionally, miR-130b-3p interacts with insulin signaling through the MAP kinase pathway. Notably, forskolin stimulation induced the translocation of DENND1A.V2 and RAB5B to the nucleus, indicating their potential direct stimulation of steroidogenesis [60]. In a separate study, Waterbury et al. investigated the relationship between ZNF217 and miR-130b-3p in PCOS patients. The study revealed that ZNF127 expression is elevated in granulosa cells and theca cells, leading to increased estradiol synthesis in the granulosa cells of healthy women. These findings suggest that ZNF217, indirectly regulated by the intermediary of miR-130b-3p, downregulates the expression of DENND1A.V2 and subsequently the expression of CYP17A1 [61]. Our miRNA profiling analysis revealed a significantly greater expression of miR-125b-5p and miR-130b-3p in enhanced exosomes than in naïve exosomes. These findings suggested that enhanced exosome therapy may have greater therapeutic efficacy than naïve exosome therapy for polycystic ovary syndrome (PCOS).

Premature ovarian insufficiency (POI) is characterized by the induction of apoptosis in granulosa cells and is a hallmark of the disease. Lv et al. reported the significant upregulation of miR-130b-3p in association with a negative correlation with PTEN expression. Similarly, miR-130b-3p was found to target and downregulate PTEN, an inhibitor of the PI3K/AKT/mTOR signaling pathway. The inhibition of PTEN by miR-130b-3p led to the activation of this pathway, ultimately suppressing apoptosis and promoting cell survival [62,63]. Moreover, the study highlighted SMAD as another target gene of miR-130b-3p. Moreover, SMAD plays a crucial role in the proliferation of granulosa cells. Consequently, the overexpression of miR-130b-3p was shown to enhance the viability and proliferation of granulosa cells, revealing the intricate molecular mechanisms contributing to the pathogenesis of POI [64].

The primary differentiation between naive and enhanced exosomes within the miRNA–protein interaction network lies in their cargo content and therapeutic potential. Naive exosomes, originating from unstimulated stem cells, harbor a diverse repertoire of miRNAs and proteins reflective of the physiological state of the producing cell. Conversely, enhanced exosomes are meticulously engineered or selectively enriched to transport specific miRNAs or proteins, customized to target precise molecular pathways implicated in disease pathogenesis.

The sorting of miRNAs into exosomes is regulated by intricate cellular mechanisms involving various RNA-binding proteins and molecular cues. For instance, the levels and phosphorylation status of Ago2, a key component of the RNA-induced silencing complex (RISC), can influence the secretion of specific miRNAs into exosomes. Additionally, RNA-binding proteins such as hnRNPA2B1 and hnRNPA1 play critical roles in recognizing and loading select miRNA species into exosomes based on sequence specificity. Furthermore, cellular factors such as the abundance of miRNAs or their target mRNAs can impact the sorting process, dictating which miRNAs are preferentially packaged into exosomes [65]. The significance of miRNA sorting into exosomes reaches beyond the internal workings of cells, encompassing communication between cells. Exosomes serve as vehicles for the transfer of functional miRNAs between cells, enabling the exchange of genetic information and regulatory signals. Upon internalization by recipient cells, exosomal miRNAs can modulate gene expression by targeting specific mRNA transcripts, thereby influencing various cellular processes and signaling pathways. This dynamic interplay between exosomal miRNAs and recipient cell proteins shapes the miRNA–protein interaction network, ultimately impacting cellular function and phenotype.

In the context of our study, the differential cargo composition of naive and enhanced exosomes is hypothesized to underlie their distinct therapeutic effects in ovarian disorders. Enhanced exosomes, enriched with specific miRNAs and proteins implicated in ovarian rejuvenation and follicular development, hold immense promise for restoring ovarian function and fertility. By precisely targeting key molecular pathways involved in ovarian dysfunction, enhanced exosomes offer a tailored therapeutic approach with the potential to mitigate disease progression and improve patient outcomes.

## 5. Conclusions

Our investigation into the proteomic and miRNA profiles of naïve exosomes and enhanced exosomes revealed significant disparities in their cargo composition, highlighting the potential of enhanced exosomes as a novel therapeutic avenue for ovarian disorders. The enhanced exosomes exhibited the elevated expression of key proteins and miRNAs implicated in various biological processes crucial for ovarian health, such as extracellular matrix maintenance, apoptosis regulation, and hormonal signaling pathways (Supplementary Table S5). The overexpression of EFEMP1, HtrA1, PAM, and SDF4 in enhanced exosomes compared to naïve exosomes underscores their potential therapeutic relevance in treating ovarian dysfunction. These proteins play vital roles in maintaining ovarian tissue integrity, regulating cellular processes, and modulating hormone levels. Moreover, the heightened expression of specific miRNAs, such as miR-1-3p, miR-103a-3p, miR-122-5p, miR-1271-5p, miR-133a-3p, miR-184, miR-203a-3p, and miR-206, in enhanced exosomes suggests their potential in targeting key pathways involved in ovarian disorders, including cell proliferation, apoptosis, and angiogenesis. In conclusion, our study sheds light on the unique cargo composition of enhanced exosomes and their therapeutic potential for diverse ovarian disorders. Future research studies will concentrate on utilizing enhanced exosomes to address a variety of ovarian disorders, tailoring the mechanism of action based on the cargo of enhanced exosomes and the distinctions between enhanced and naive exosomes.

## Data Availability

The raw data supporting the conclusions of this article will be made available by the authors on request.

## Supplementary Materials

The following supporting information can be downloaded at: https://www.mdpi.com/article/10.3390/jpm14050482/s1, Table S1: Significantly overexpressed proteins in enhanced exosomes compared to naïve exosomes across all categories (all proteins, extracellular vesicle, and exosome), including fold change analysis; Table S2: KEGG pathway analysis of proteins overexpressed in enhanced exosomes compared to naïve exosomes; Table S3: Complete list of upregulated and downregulated miRNA in enhanced exosomes compared to naïve exosomes, including fold change and *p*-value; Table S4: Target genes of each miRNA; Table S5: Enhanced exosome cargo and therapeutic mechanism in ovarian disorders; Figure S1: The expression of exosomal markers; Figure S2: Network of Known miRNAs and Their Target Genes.
