# Supplementary material for: A Comparative Analysis of Naïve Exosomes and Enhanced Exosomes with a Focus on the Treatment Potential in Ovarian Disorders
Source: J Pers Med. 2024 Apr 30;14(5):482. doi: 10.3390/jpm14050482 (PMC11122298; doi:10.3390/jpm14050482)
Supplement: Supplementary file 1 [file jpm-14-00482-s001.zip › supplementary data 2/Supplementary data, Table S1.pdf]

No	Name	Fold change	No	Name	Fold change	No	Name	Fold change
All Proteins								
**1**	HIST2H3A	5.415774	**43**	CAPRIN1	2.030893	**85**	ILF3	1.574275
**2**	LAMA5	4.912965	**44**	USP15	1.99445	**86**	MATR3	1.573667
**3**	APLP2	4.839289	**45**	SPTAN1	1.946298	**87**	MRC2	1.567592
**4**	FXR1	4.057065	**46**	PXDN	1.927531	**88**	PSMG1	1.542099
**5**	EFEMP1	3.848948	**47**	FKBP9	1.915003	**89**	TUBAL3	1.541929
**6**	SF3B3	3.700617	**48**	AP1M1	1.902163	**90**	ACTG1	1.537505
**7**	HTRA1	3.521209	**49**	EIF4G1	1.879045	**91**	TPM2	1.524892
**8**	UBA2	3.384767	**50**	RAB2A	1.874018	**92**	PTBP1	1.522484
**9**	MMP19	3.333479	**51**	NXN	1.86393	**93**	SEC23A	1.522406
**10**	SRPX	3.321563	**52**	DDX39A	1.863168	**94**	PTPN1	1.517119
**11**	HMGA2	3.281848	**53**	NUDT21	1.850507	**95**	NID2	1.514909
**12**	PTGES3	3.264797	**54**	AARS	1.835753	**96**	GLS	1.513476
**13**	PREP	3.107649	**55**	LRRC17	1.804368	**97**	SF3B1	1.502484
**14**	MGAT5	2.894506	**56**	PSME3	1.801918	**98**	XRCC5	1.502248
**15**	RHOA	2.882499	**57**	IAH1	1.797593	**99**	SPTBN1	1.500168
**16**	ADD3	2.85615	**58**	CRTAP	1.788128	**100**	NUP93	1.494096
**17**	CDC42BPB	2.853821	**59**	MFAP4	1.785901	**101**	YWHAE	1.489433
**18**	UNC45A	2.850593	**60**	NUCB1	1.783986	**102**	ECH1	1.487053
**19**	CDH23	2.80839	**61**	HNRNPR	1.776111	**103**	SPON2	1.468898
**20**	PGAM5	2.806167	**62**	UGGT1	1.763243	**104**	NUP98	1.466304
**21**	PAM	2.766931	**63**	POSTN	1.758704	**105**	PFKM	1.461211
**22**	RSU1	2.674164	**64**	SEC24D	1.75617	**106**	EEF1D	1.44615
**23**	ARL1	2.633532	**65**	DRG2	1.750284	**107**	ADSL	1.431011
**24**	SNRPD1	2.589605	**66**	ARMT1	1.74827	**108**	RPS24	1.428671
**25**	GNPAT	2.537942	**67**	TOP1	1.733486	**109**	CAPN2	1.425369
**26**	MCM4	2.492846	**68**	RCN1	1.729342	**110**	NAP1L1	1.411412
**27**	SDF4	2.474751	**69**	TIMM44	1.722561	**111**	LTBP1	1.402398
**28**	SMARCA5	2.467528	**70**	ALDH9A1	1.711249	**112**	FBLN1	1.392655
**29**	HEATR1	2.418155	**71**	UGP2	1.70947	**113**	SYNCRIP	1.391316
**30**	MCM2	2.387727	**72**	NUP133	1.707811	**114**	FKBP11	1.390567
**31**	ANTXR1	2.379471	**73**	CLU	1.706395	**115**	HNRNPA1	1.383585
**32**	PRKAG1	2.320002	**74**	NACA	1.704063	**116**	HNRNPA3	1.376628
**33**	TRAP1	2.287293	**75**	CSE1L	1.701936	**117**	HNRNPK	1.375381
**34**	EIF3H	2.207335	**76**	AGRN	1.694546	**118**	EMILIN1	1.372395
**35**	RPS15	2.185175	**77**	GBE1	1.691186	**119**	CLSTN1	1.370861
**36**	MATN3	2.174195	**78**	CCAR2	1.688942	**120**	IFI16	1.355165
**37**	MATN2	2.14227	**79**	MTDH	1.68379	**121**	SERPINF1	1.35243
**38**	TOP2B	2.130412	**80**	COPE	1.683695	**122**	HADHA	1.349318
**39**	BTF3	2.125791	**81**	PSME1	1.675359	**123**	CNN2	1.347437
**40**	CHD4	2.092596	**82**	NAMPT	1.654905	**124**	FASN	1.341763
**41**	PRMT1	2.081544	**83**	MLEC	1.644746	**125**	RPS7	1.336084
**42**	NDUFA12	2.057606	**84**	GOLT1B	1.599967	**126**	NCKAP1	1.327883

**127**	LAP3	1.324775	**149**	EIF3CL	1.200711	**171**	ACO2	1.064816
**128**	SEC61G	1.324226	**150**	COMT	1.19536	**172**	CCDC148	1.061061
**129**	XRCC6	1.323747	**151**	DDX5	1.191062	**173**	COPS3	1.055834
**130**	EIF2S3	1.292177	**152**	STOML2	1.174188	**174**	TGFBI	1.049859
**131**	PDLIM5	1.286318	**153**	MAN2A1	1.171845	**175**	PSMD5	1.049785
**132**	NCL	1.285363	**154**	SRP9	1.164959	**176**	CAPN1	1.049089
**133**	RBBP7	1.281747	**155**	LMNA	1.156099	**177**	GDI2	1.047599
**134**	NT5DC2	1.280941	**156**	HSPG2	1.14336	**178**	TM9SF2	1.047567
**135**	TIA1	1.256574	**157**	CCT5	1.13923	**179**	PGM3	1.041765
**136**	MAMDC2	1.253956	**158**	KARS	1.135454	**180**	SPOCK1	1.039038
**137**	LARS	1.249013	**159**	EEF2	1.130744	**181**	EPRS	1.033018
**138**	HSPH1	1.240383	**160**	SRSF1	1.129664	**182**	COL14A1	1.031871
**139**	THBS2	1.239299	**161**	COPB1	1.102414	**183**	NTMT1	1.029058
**140**	TOMM70	1.235044	**162**	IPO5	1.101549	**184**	AEBP1	1.025879
**141**	PCOLCE	1.233321	**163**	TCP1	1.094213	**185**	SERPINH1	1.025467
**142**	QPCT	1.23047	**164**	SDHB	1.076817	**186**	ASPH	1.024545
**143**	ATAD3A	1.223842	**165**	PDIA6	1.074409	**187**	COPS2	1.022048
**144**	TTLL12	1.21968	**166**	HNRNPA2B1	1.073607	**188**	RBM39	1.019784
**145**	TRA2B	1.218602	**167**	RPLP2	1.072141	**189**	EIF2S1	1.014637
**146**	EEF1G	1.214396	**168**	DDX3X	1.067722	**190**	SNX4	1.011258
**147**	SRI	1.212749	**169**	NID1	1.067117	**191**	IMPDH2	1.003026
**148**	CBR1	1.205816	**170**	GFPT1	1.065445			
**Extracellular vesicle Proteins**								
**1**	HIST2H3A	5.415774	**24**	NUCB1	1.783985	**46**	FBLN1	1.39266
**2**	LAMA5	4.91297	**25**	UGGT1	1.763242	**47**	HNRNPA1	1.383585
**3**	APLP2	4.839288	**26**	POSTN	1.758705	**48**	HNRNPK	1.375375
**4**	EFEMP1	3.848946	**27**	ALDH9A1	1.711245	**49**	EMILIN1	1.372395
**5**	HTRA1	3.521209	**28**	UGP2	1.70947	**50**	CLSTN1	1.37086
**7**	MMP19	3.333482	**29**	CLU	1.706395	**51**	SERPINF1	1.35243
**8**	MGAT5	2.894505	**30**	CSE1L	1.70194	**52**	CNN2	1.347439
**9**	RHOA	2.882499	**31**	AGRN	1.694545	**53**	FASN	1.341765
**10**	CDC42BPB	2.853816	**32**	GBE1	1.691186	**54**	NCKAP1	1.327882
**11**	PAM	2.766933	**33**	PSME1	1.675363	**55**	LAP3	1.324778
**12**	RSU1	2.674161	**34**	NAMPT	1.654908	**56**	XRCC6	1.323745
**13**	SDF4	2.474751	**35**	ILF3	1.574274	**57**	EIF2S3	1.292175
**14**	EIF3H	2.207334	**36**	ACTG1	1.537505	**58**	NCL	1.285365
**15**	MATN3	2.174198	**37**	PTBP1	1.522484	**59**	MAMDC2	1.253958
**16**	MATN2	2.142269	**38**	NID2	1.51491	**60**	HSPH1	1.240385
**17**	SPTAN1	1.946295	**39**	XRCC5	1.50225	**61**	THBS2	1.239295
**18**	PXDN	1.927535	**40**	SPTBN1	1.500175	**62**	TOMM70	1.235046
**19**	AP1M1	1.90216	**41**	YWHAE	1.489435	**63**	PCOLCE	1.23332
**20**	RAB2A	1.874022	**42**	ECH1	1.487053	**64**	QPCT	1.230471
**21**	AARS	1.835753	**43**	SPON2	1.468895	**65**	EEF1G	1.2144
**22**	CRTAP	1.804366	**44**	CAPN2	1.42537	**66**	SRI	1.21275
**23**	MFAP4	1.788129	**45**	LTBP1	1.402395	**67**	CBR1	1.205814

**68**	COMT	1.19536	**76**	PDIA6	1.07441	**84**	GDI2	1.0476
**69**	DDX5	1.19106	**77**	HNRNPA2B1	1.07361	**85**	TM9SF2	1.047571
**70**	MAN2A1	1.171845	**78**	RPLP2	1.072145	**86**	SPOCK1	1.03904
**71**	HSPG2	1.14336	**79**	DDX3X	1.06772	**87**	COL14A1	1.031875
**72**	CCT5	1.13923	**80**	NID1	1.06712	**88**	AEBP1	1.025875
**73**	KARS	1.135458	**81**	GFPT1	1.06544	**89**	SERPINH1	1.025465
**74**	EEF2	1.13075	**82**	TGFBI	1.049855	**90**	EIF2S1	1.014636
**75**	TCP1	1.09421	**83**	CAPN1	1.04909	**91**	IMPDH2	1.003025
**Exosome Proteins**								
**1**	HIST2H3A	5.415775	**25**	GBE1	1.691186	**49**	QPCT	1.230471
**2**	LAMA5	4.91297	**26**	PSME1	1.675363	**50**	EEF1G	1.2144
**3**	APLP2	4.839288	**27**	NAMPT	1.654908	**51**	SRI	1.21275
**4**	EFEMP1	3.848946	**28**	ACTG1	1.537505	**52**	CBR1	1.205814
**5**	HTRA1	3.521209	**29**	PTBP1	1.522484	**53**	COMT	1.19536
**6**	MGAT5	2.894505	**30**	NID2	1.51491	**54**	DDX5	1.19106
**7**	RHOA	2.882499	**31**	SPTBN1	1.500175	**55**	MAN2A1	1.171845
**8**	CDC42BPB	2.853816	**32**	YWHAE	1.489435	**56**	HSPG2	1.14336
**9**	PAM	2.766933	**33**	ECH1	1.487053	**57**	CCT5	1.13923
**10**	RSU1	2.674161	**34**	SPON2	1.468895	**58**	EEF2	1.13075
**11**	SDF4	2.474751	**35**	CAPN2	1.42537	**59**	TCP1	1.09421
**12**	EIF3H	2.207334	**36**	FBLN1	1.39266	**60**	PDIA6	1.07441
**13**	SPTAN1	1.946295	**37**	HNRNPA1	1.383585	**61**	HNRNPA2B1	1.07361
**14**	PXDN	1.927535	**38**	HNRNPK	1.375375	**62**	RPLP2	1.072145
**15**	AP1M1	1.90216	**39**	EMILIN1	1.372395	**63**	DDX3X	1.06772
**16**	RAB2A	1.874022	**40**	SERPINF1	1.35243	**64**	NID1	1.06712
**17**	AARS	1.835753	**41**	FASN	1.341765	**65**	GFPT1	1.06544
**18**	NUCB1	1.783985	**42**	NCKAP1	1.327882	**66**	TGFBI	1.049855
**19**	UGGT1	1.763242	**43**	LAP3	1.324778	**67**	CAPN1	1.04909
**20**	ALDH9A1	1.711245	**44**	EIF2S3	1.292175	**68**	GDI2	1.0476
**21**	UGP2	1.70947	**45**	NCL	1.285365	**69**	TM9SF2	1.047571
**22**	CLU	1.706395	**46**	HSPH1	1.240385	**70**	AEBP1	1.025875
**23**	CSE1L	1.70194	**47**	TOMM70	1.235046	**71**	EIF2S1	1.014636
**24**	AGRN	1.694545	**48**	PCOLCE	1.23332	**72**	IMPDH2	1.003025
