# Supplementary material for: A Comparative Analysis of Naïve Exosomes and Enhanced Exosomes with a Focus on the Treatment Potential in Ovarian Disorders
Source: J Pers Med. 2024 Apr 30;14(5):482. doi: 10.3390/jpm14050482 (PMC11122298; doi:10.3390/jpm14050482)
Supplement: Supplementary file 1 [file jpm-14-00482-s001.zip › supplementary data 2/Supplementary data, Table S2.pdf]

Name	KEGG Pathways
**HIST2H3A**	Alcoholism; Systemic lupus erythematosus; Transcriptional misregulation in cancer
**LAMA5**	Amoebiasis; ECM-receptor interaction; Focal adhesion; Human papillomavirus infection; Pathways in cancer; PI3K-Akt signaling pathway; Small cell lung cancer; Toxoplasmosis
**FXR1**	RNA transport
**SF3B3**	Spliceosome
**UBA2**	Ubiquitin mediated proteolysis
**HMGA2**	MicroRNAs in cancer; Transcriptional misregulation in cancer
**PTGES3**	Arachidonic acid metabolism; Metabolic pathways
**PREP**	Renin-angiotensin system
**MGAT5**	Metabolic pathways; N-Glycan biosynthesis
**RHOA**	Adherens junction; Axon guidance; Bacterial invasion of epithelial cells; cAMP signaling pathway; cGMP-PKG signaling pathway; Chemokine signaling pathway; Colorectal cancer; C-type lectin receptor signaling pathway; Endocytosis; Fluid shear stress and atherosclerosis; Focal adhesion; Human cytomegalovirus infection; Leukocyte transendothelial migration; MicroRNAs in cancer; mTOR signaling pathway; Neurotrophin signaling pathway; NOD-like receptor signaling pathway; Oxytocin signaling pathway; Pancreatic secretion; Parathyroid hormone synthesis, secretion and action; Pathogenic Escherichia coli infection; Pathways in cancer; Pertussis; Phospholipase D signaling pathway; Platelet activation; Proteoglycans in cancer;Rap1 signaling pathway; Ras signaling pathway; Regulation of actin cytoskeleton; Sphingolipid signaling pathway; T cell receptor signaling pathway; TGF-beta signaling pathway; Tight junction; Tuberculosis; Vascular smooth muscle contraction; Viral carcinogenesis; Wnt signaling pathway; Yersinia infection
**PGAM5**	Mitophagy - animal; Necroptosis; TNF signaling pathway
**SNRPD1**	Spliceosome; Systemic lupus erythematosus
**GNPAT**	Glycerophospholipid metabolism; Peroxisome
**MCM4**	Cell cycle; DNA replication
**HEATR1**	Ribosome biogenesis in eukaryotes
**MCM2**	Cell cycle; DNA replication
**ANTXR1**	NOD-like receptor signaling pathway
**PRKAG1**	Adipocytokine signaling pathway; AMPK signaling pathway; Apelin signaling pathway; Circadian rhythm; FoxO signaling pathway; Glucagon signaling pathway; Hypertrophic cardiomyopathy (HCM); Insulin resistance; Insulin signaling pathway; Longevity regulating pathway; Longevity regulating pathway - multiple species; Non-alcoholic fatty liver disease (NAFLD); Oxytocin signaling pathway; Thermogenesis; Tight junction
**EIF3H**	Measles; RNA transport
**RPS15**	Ribosome
**TOP2B**	Platinum drug resistance
**CHD4**	Human papillomavirus infection; Viral carcinogenesis
**PRMT1**	FoxO signaling pathway; Glucagon signaling pathway

**NDUFA12**	Alzheimer disease; Huntington disease; Metabolic pathways; Non-alcoholic fatty liver disease (NAFLD); Oxidative phosphorylation; Parkinson disease; Retrograde endocannabinoid signaling; Thermogenesis
**USP15**	Mitophagy - animal
**SPTAN1**	Apoptosis
**AP1M1**	Human immunodeficiency virus 1 infection; Lysosome
**EIF4G1**	RNA transport; Viral myocarditis
**RAB2A**	AMPK signaling pathway
**NUDT21**	mRNA surveillance pathway
**AARS**	Aminoacyl-tRNA biosynthesis
**PSME3**	Antigen processing and presentation; Hepatitis C; Proteasome
**UGGT1**	Protein processing in endoplasmic reticulum
**SEC24D**	Protein processing in endoplasmic reticulum
**ALDH9A1**	Arginine and proline metabolism; Ascorbate and aldarate metabolism;beta-Alanine metabolism; Fatty acid degradation; Glycerolipid metabolism; Glycolysis / Gluconeogenesis; Histidine metabolism; Lysine degradation; Metabolic pathways; Pyruvate metabolism; Tryptophan metabolism; Valine, leucine and isoleucine degradation
**UGP2**	Amino sugar and nucleotide sugar metabolism; Galactose metabolism; Metabolic pathways; Pentose and glucuronate interconversions; Starch and sucrose metabolism
**NUP133**	RNA transport
**CLU**	Complement and coagulation cascades
**AGRN**	ECM-receptor interaction
**GBE1**	Metabolic pathways; Starch and sucrose metabolism
**PSME1**	Antigen processing and presentation; Proteasome
**NAMPT**	Metabolic pathways; Nicotinate and nicotinamide metabolism; NOD-like receptor signaling pathway
**MRC2**	Phagosome; Tuberculosis
**TUBAL3**	Apoptosis; Gap junction; Pathogenic Escherichia coli infection; Phagosome; Tight junction
**ACTG1**	Adherens junction; Apoptosis; Arrhythmogenic right ventricular cardiomyopathy (ARVC);Bacterial invasion of epithelial cells; Dilated cardiomyopathy (DCM);Fluid shear stress and atherosclerosis; Focal adhesion; Hepatocellular carcinoma; Hippo signaling pathway; Hypertrophic cardiomyopathy (HCM);Influenza A; Leukocyte transendothelial migration; Oxytocin signaling pathway; Pathogenic Escherichia coli infection; Phagosome; Platelet activation; Proteoglycans in cancer;Rap1 signaling pathway; Regulation of actin cytoskeleton; Salmonella infection; Shigellosis; Thermogenesis; Thyroid hormone signaling pathway; Tight junction; Vibrio cholerae infection; Viral myocarditis; Yersinia infection
**TPM2**	Adrenergic signaling in cardiomyocytes; Cardiac muscle contraction; Dilated cardiomyopathy (DCM); Hypertrophic cardiomyopathy (HCM)
**SEC23A**	Protein processing in endoplasmic reticulum
**PTPN1**	Adherens junction; Insulin resistance; Insulin signaling pathway

**GLS**	Alanine, aspartate and glutamate metabolism; Arginine biosynthesis; Central carbon metabolism in cancer; D-Glutamine and D-glutamate metabolism; GABAergic synapse; Glutamatergic synapse; Metabolic pathways; MicroRNAs in cancer; Proximal tubule bicarbonate reclamation
**SF3B1**	Spliceosome
**XRCC5**	Non-homologous end-joining
**NUP93**	RNA transport
**YWHAE**	Cell cycle; Hepatitis C; Hippo signaling pathway; Neurotrophin signaling pathway; Oocyte meiosis; PI3K-Akt signaling pathway; Viral carcinogenesis
**ECH1**	Peroxisome
**NUP98**	Influenza A;RNA transport
**PFKM**	AMPK signaling pathway; Biosynthesis of amino acids; Carbon metabolism; Central carbon metabolism in cancer; Fructose and mannose metabolism; Galactose metabolism; Glucagon signaling pathway; Glycolysis / Gluconeogenesis; HIF-1 signaling pathway; Metabolic pathways; Pentose phosphate pathway; RNA degradation
**ADSL**	Alanine, aspartate and glutamate metabolism; Metabolic pathways; Purine metabolism
**RPS24**	Ribosome
**CAPN2**	Alzheimer disease; Apoptosis; Cellular senescence; Focal adhesion; Necroptosis; Protein processing in endoplasmic reticulum
**LTBP1**	TGF-beta signaling pathway
**HNRNPA1**	Spliceosome
**HNRNPA3**	Spliceosome
**HNRNPK**	MicroRNAs in cancer; Sliceosome; Viral carcinogenesis
**IFI16**	NOD-like receptor signaling pathway
**SERPINF1**	Wnt signaling pathway
**HADHA**	beta-Alanine metabolism; Butanoate metabolism; Carbon metabolism; Fatty acid degradation; Fatty acid elongation; Fatty acid metabolism; Lysine degradation; Metabolic pathways; Propanoate metabolism; Tryptophan metabolism; Valine, leucine and isoleucine degradation
**FASN**	AMPK signaling pathway; Fatty acid biosynthesis; Fatty acid metabolism; Insulin signaling pathway; Metabolic pathways
**RPS7**	Ribosome
**LAP3**	Arginine and proline metabolism; Glutathione metabolism; Metabolic pathways
**SEC61G**	Phagosome; Protein export; Protein processing in endoplasmic reticulum; Vibrio cholerae infection
**XRCC6**	Non-homologous end-joining
**EIF2S3**	RNA transport
**NCKAP1**	Regulation of actin cytoskeleton
**NCL**	Pathogenic Escherichia coli infection
**LARS**	Aminoacyl-tRNA biosynthesis
**HSPH1**	Protein processing in endoplasmic reticulum
**THBS2**	ECM-receptor interaction; Focal adhesion; Human papillomavirus infection;Malaria; Phagosome;PI3K-Akt signaling pathway

**TRA2B**	Spliceosome
**EEF1G**	Legionellosis
**CBR1**	Arachidonic acid metabolism; Chemical carcinogenesis; Folate biosynthesis; Metabolic pathways; Metabolism of xenobiotics by cytochrome P450
**EIF3CL**	RNA transport
**COMT**	Dopaminergic synapse; Metabolic pathways; Steroid hormone biosynthesis; Tyrosine metabolism
**DDX5**	Proteoglycans in cancer; Spliceosome; Transcriptional misregulation in cancer
**MAN2A1**	Metabolic pathways; N-Glycan biosynthesis
**SRP9**	Protein export
**LMNA**	Apoptosis; Arrhythmogenic right ventricular cardiomyopathy (ARVC); Dilated cardiomyopathy (DCM);Hypertrophic cardiomyopathy (HCM)
**HSPG2**	ECM-receptor interaction; Hepatitis B; Proteoglycans in cancer
**KARS**	Aminoacyl-tRNA biosynthesis
**EEF2**	AMPK signaling pathway; Oxytocin signaling pathway
**SRSF1**	Herpes simplex virus 1 infection; IL-17 signaling pathway; Spliceosome
**SDHB**	Alzheimer disease; Carbon metabolism; Citrate cycle (TCA cycle); Huntington disease; Metabolic pathways; Non-alcoholic fatty liver disease (NAFLD);Oxidative phosphorylation; Parkinson disease; Thermogenesis
**PDIA6**	Protein processing in endoplasmic reticulum
**RPLP2**	Ribosome
**DDX3X**	Hepatitis B;RIG-I-like receptor signaling pathway; Viral carcinogenesis
**GFPT1**	Alanine, aspartate and glutamate metabolism; Amino sugar and nucleotide sugar metabolism; Insulin resistance; Metabolic pathways
**ACO2**	2-Oxocarboxylic acid metabolism; Biosynthesis of amino acids; Carbon metabolism; Citrate cycle (TCA cycle); Glyoxylate and dicarboxylate metabolism; Metabolic pathways
**CAPN1**	Alzheimer disease; Apoptosis; Cellular senescence; Necroptosis; Protein processing in endoplasmic reticulum
**PGM3**	Amino sugar and nucleotide sugar metabolism; Metabolic pathways
**EPRS**	Aminoacyl-tRNA biosynthesis; Metabolic pathways; Porphyrin and chlorophyll metabolism
**COL14A1**	Protein digestion and absorption
**ASPH**	Calcium signaling pathway; Cardiac muscle contraction
**EIF2S1**	Apoptosis; Autophagy - animal; Hepatitis C; Herpes simplex virus 1 infection; Influenza A; Measles; Non-alcoholic fatty liver disease (NAFLD); Protein processing in endoplasmic reticulum; RNA transport
**SNX4**	Endocytosis
**IMPDH2**	Drug metabolism - other enzymes; Metabolic pathways; Purine metabolism
