# Supplementary material for: A Comparative Analysis of Naïve Exosomes and Enhanced Exosomes with a Focus on the Treatment Potential in Ovarian Disorders
Source: J Pers Med. 2024 Apr 30;14(5):482. doi: 10.3390/jpm14050482 (PMC11122298; doi:10.3390/jpm14050482)
Supplement: Supplementary file 1 [file jpm-14-00482-s001.zip › supplementary data 2/Supplementary data, Table S3.pdf]

No	Name	Fold change	*P-Value*
		**Upregulation**	
**1**	hsa-miR-1-3p	3.59172791	3.98E-14
**2**	hsa-miR-10399-5p	2.87348255	0.00082998
**3**	hsa-miR-103a-3p	1.49383785	0.00037265
**4**	hsa-miR-103b	1.49336934	0.00037426
**5**	hsa-miR-106a-5p	3.24158369	0.00136958
**6**	hsa-miR-122-5p	1.5476995	0.00473977
**7**	hsa-miR-122b-3p	1.5476995	0.00473977
**8**	hsa-miR-125b-2-3p	2.14979422	0.002639
**9**	hsa-miR-125b-5p	1.22773021	0.00340961
**10**	hsa-miR-1271-5p	1.38021558	0.00217771
**11**	hsa-miR-130b-3p	2.47667999	8.36E-08
**12**	hsa-miR-133a-3p	7.81705346	1.61E-06
**13**	hsa-miR-133a-5p	6.75988533	0.00085757
**14**	hsa-miR-183-5p	2.17351674	3.43E-06
**15**	hsa-miR-184	7.00171563	2.55E-16
**16**	hsa-miR-203a-3p	2.21892924	0.00214237
**17**	hsa-miR-203b-5p	2.21892924	0.00214237
**18**	hsa-miR-206	6.88430644	0.00051727
**19**	hsa-miR-224-5p	3.1203814	1.71E-11
**20**	hsa-miR-3177-3p	3.4632814	5.75E-05
**21**	hsa-miR-335-5p	1.18548323	0.00502854
**22**	hsa-miR-452-5p	4.94481552	1.01E-21
**23**	hsa-miR-4802-3p	6.75988533	0.00085757
**24**	hsa-miR-486-3p	3.30622943	3.86E-10
**25**	hsa-miR-486-5p	3.4069645	1.13E-10
**26**	hsa-miR-551a	6.75988533	0.00085757
**27**	hsa-miR-584-5p	2.22954966	2.75E-06
**28**	hsa-miR-6500-3p	7.75321388	3.61E-06
**29**	hsa-miR-675-3p	4.33704987	7.55E-05
**30**	hsa-miR-6842-3p	5.14046577	2.40E-07
**31**	hsa-miR-760	2.30573093	0.00390492
**32**	hsa-miR-9-5p	2.59845073	3.44E-06
**33**	hsa-miR-92b-5p	2.80344337	0.0011736
**34**	hsa-miR-99a-5p	2.35076907	5.01E-08
		**Downregulation**	
**1**	hsa-let-7e-3p	-8.1234092	8.20E-08
**2**	hsa-let-7g-5p	-1.2092036	0.00365202
**3**	hsa-miR-103a-2-5p	-2.6281159	2.97E-05
**4**	hsa-miR-1260b	-2.3024136	0.00390492
**5**	hsa-miR-1304-5p	-2.1843225	3.51E-05
**6**	hsa-miR-130a-5p	-4.8633752	9.42E-10
**7**	hsa-miR-136-3p	-1.520873	0.00062351
**8**	hsa-miR-145-5p	-1.2130305	0.00574006

**9**	hsa-miR-148a-3p	-2.4151652	2.27E-08
**10**	hsa-miR-151a-3p	-1.4835195	0.00040116
**11**	hsa-miR-197-3p	-1.9085419	0.00021354
**12**	hsa-miR-199a-5p	-1.389932	0.00094196
**13**	hsa-miR-200b-3p	-2.1137361	0.00021171
**14**	hsa-miR-200c-3p	-2.5358528	5.44E-06
**15**	hsa-miR-205-5p	-2.2679954	0.00049824
**16**	hsa-miR-218-5p	-1.2356346	0.00327085
**17**	hsa-miR-219a-5p	-6.6816874	0.00085757
**18**	hsa-miR-219b-3p	-6.6816874	0.00085757
**19**	hsa-miR-222-3p	-1.1822339	0.00455561
**20**	hsa-miR-24-2-5p	-2.092012	1.43E-06
**21**	hsa-miR-2682-5p	-2.2881239	1.31E-07
**22**	hsa-miR-26a-1-3p	-6.6816874	0.00085757
**23**	hsa-miR-29a-5p	-1.5854265	0.00214202
**24**	hsa-miR-30b-3p	-6.8606054	0.00031521
**25**	hsa-miR-30e-3p	-1.5770671	0.00142297
**26**	hsa-miR-3117-3p	-1.6368347	0.00084628
**27**	hsa-miR-3138	-2.1914474	0.00281747
**28**	hsa-miR-3158-3p	-1.5523796	0.00431386
**29**	hsa-miR-337-3p	-1.3330192	0.00350524
**30**	hsa-miR-34c-5p	-1.4472651	0.00074061
**31**	hsa-miR-3605-5p	-1.5521457	0.00231836
**32**	hsa-miR-365a-5p	-1.6039386	0.00231248
**33**	hsa-miR-3688-3p	-6.3632685	0.00417417
**34**	hsa-miR-372-3p	-6.8606054	0.00031521
**35**	hsa-miR-375-3p	-3.2418091	1.03E-06
**36**	hsa-miR-377-3p	-2.0711849	0.00102772
**37**	hsa-miR-381-3p	-1.9667295	3.76E-06
**38**	hsa-miR-410-3p	-1.7821089	0.00015804
**39**	hsa-miR-431-5p	-2.1771304	1.28E-06
**40**	hsa-miR-4504	-7.2297499	4.78E-05
**41**	hsa-miR-451a	-2.1905684	0.00012861
**42**	hsa-miR-455-5p	-1.2812281	0.00218672
**43**	hsa-miR-4775	-2.2062249	0.00040748
**44**	hsa-miR-487b-3p	-1.7527052	4.76E-05
**45**	hsa-miR-493-3p	-1.9691019	3.75E-06
**46**	hsa-miR-493-5p	-1.2447617	0.00285821
**47**	hsa-miR-495-3p	-1.6327052	0.00012179
**48**	hsa-miR-516a-5p	-8.933553	5.13E-10
**49**	hsa-miR-516b-5p	-6.8731909	2.64E-16
**50**	hsa-miR-517a-3p	-8.6711086	7.10E-09
**51**	hsa-miR-518a-3p	-6.477397	0.00243493
**52**	hsa-miR-518b	-3.735307	0.00236482
**53**	hsa-miR-518c-3p	-7.0932073	0.0001205

**54**	hsa-miR-518e-3p	-6.477397	0.00243493
**55**	hsa-miR-519c-5p	-7.3544796	1.96E-05
**56**	hsa-miR-520a-3p	-7.0932073	0.0001205
**57**	hsa-miR-520g-3p	-6.7739182	0.00051726
**58**	hsa-miR-526b-5p	-7.6746435	2.40E-06
**59**	hsa-miR-532-5p	-1.3262062	0.0016618
**60**	hsa-miR-548aq-3p	-4.4682404	2.88E-05
**61**	hsa-miR-548ar-3p	-2.1302381	0.00011466
**62**	hsa-miR-548g-5p	-4.4682404	2.88E-05
**63**	hsa-miR-548o-3p	-1.7487355	8.64E-05
**64**	hsa-miR-548u	-4.0956384	0.00034853
**65**	hsa-miR-582-3p	-2.6527992	3.87E-07
**66**	hsa-miR-641	-1.8728469	0.00036298
**67**	hsa-miR-654-5p	-1.366141	0.00175821
**68**	hsa-miR-708-3p	-1.5201664	0.00214422
**69**	hsa-miR-7704	-2.9962217	5.36E-05
**70**	hsa-miR-889-3p	-1.3915236	0.00131223
**71**	novel_215	-4.3395251	8.17E-05
**72**	novel_244	-6.6816874	0.00085757
