# Supplementary material for: A Comparative Analysis of Naïve Exosomes and Enhanced Exosomes with a Focus on the Treatment Potential in Ovarian Disorders
Source: J Pers Med. 2024 Apr 30;14(5):482. doi: 10.3390/jpm14050482 (PMC11122298; doi:10.3390/jpm14050482)
Supplement: Supplementary file 1 [file jpm-14-00482-s001.zip › supplementary data 2/Supplementary data, Table S4.pdf]

miRNAs	Targets
hsa-miR-103a-3p	IL32,P4HA2,DRD4,TMEM101,TEKT2,PPP6R2,PLEK2,CHGA,USP11,PEX11G,NOD1,HSD17B1,PPY,OSCP1,TBX4,FBXO24,POLM,ITPR2,UBE2C,WFDC3,PACSIN1,DDX49,ANGPTL8,MYBPH,MORC4,OS9,SQOR,SLC3A1,VSX2,CCNE1,MOV10L1,SIRPB1,SEMA3A,HPX,ETNK1,ITGAX,NDUFB10,DEF8,ZNF750,SIK1,SLC44A3,REG3G,GPR6,HIKESHI,CC2D1B,OBSCN,GSTT2B,SPON2,PIP,SSU72,OLFML2B,BOLA3,ZDHHC19,FBXW12,SERPINA4,TMED3,PHB,DEFB4A,WDR48,UMOD,ANKS3,DISP3,EXOSC10,PTCRA,REG3A,MTCL1,C17orf62,AVEN,NT5DC2,MYEOV,CEP290,ALAS1,MADD,SLC22A8,HMGA1,MCTP1,RPS6KB2,ST8SIA5,DMAP1,CCNG2,DEFB4B,SLC25A20,TMEM184A,SLC26A10,SPACA4,ZNF491,CD37,PRMT5,CYP4F12,IL32,KDSR,CTAG1B,TRMT12,CLEC4G,SLC52A2,ANKRD11,TMEM89,NAT8L,ACSM5,PGP,CCDC84,VSTM2B,FRMD1,ZFYVE19,ESR1,NAPRT,TBC1D16,OAS2,CD55,SLC35E4,CASZ1,PCNX2,SDSL,ZNF683,GPAA1,KEL,OR8B4,XAB2,KCNN3,LFNG,NIF3L1,MAP4,DISP1,FLNA,TEDC2,SCAMP5,GLMP,TPR,LY9,CD1E,SH2D2A,KHDC4,CALHM6,SELENBP1,ACADSB,RPRD2,LDOC1,ZDHHC16,DIRAS3,SLC29A1,RAPGEF1,CD40,YIPF3,TFAP2E,TMEM35B,TGIF2,MFSD14C,SP5,WDR45,SPATA31A7,BTN3A2,PCDHA4,SEPT8,DCTN3,TTLL10,AVP,SLC22A23,ARHGAP6,MTUS2,SLC13A5,ITSN1,TOM1,ZG16B,CTBP1,ARNTL,KIF19,SIGLEC6,AKT1S1,FCRL6,SORBS2,ANKRD39,SEMA5B,VKORC1,IZUMO4,WDR6,AKAP10,CCDC51,KIR2DL4,DDR1,RAB2A,CHMP1A,TREH,GSTP1,MS4A18,ANO7,NAGA,C2orf73,RHBDD3,RPS14,PIGQ,KCNC4,ADPRHL1,CACNA1B,CHN1,STK11IP,SLC2A6,KIAA0930,A1CF,PGC,FAM229A,IFRD2,SLC2A8,CD6,THNSL2,IFT172,C3orf18,MRPL10,HYAL2,KBTBD2,PSPH,BCS1L,PLA2G6,PHF1,SLCO2B1,PIGV,SENP2,ARHGEF33,SRPK3,ZNF485,RLN3,VAMP8,CLCN1,RNF144A,PSMD2,PFKP,COL6A3,HJURP,SLC12A9,EDEM1,EPM2A,HAUS7,TRIM26,NCDN,SNRK,TST,ZNF382,FBXO34,PDE2A,C16orf91,SEMA3E,FAM90A1,FOLR3,GTF2I,OPN4,OR10C1,CCDC188,BCAS4,GSDME,CCT3,NR5A2,MRPL28,ZNF185,TTC22,ORC5,MBOAT7,TMEM150A,ANAPC1,C2orf92,DOT1L,RBKS,NDUFS8,FAM126B,CLCN4,FOLR2,PDE3B,DCAF6,NELFA,PCSK5,SCMH1,GOLGA2,IRF1,XPNPEP1,KLHDC8A,ATP11C,CFLAR,PPP2R5D,TNNC1,C1orf116,TFPI2,SLC25A48,CBS,PPP1R16B,ALAS2,D2HGDH,ASCC2,MRPS5,PRKD3,PGAP2,IL1RAP,DEGS1,RABL6,PYCR2,GPR157,PLP1,SYP,CYP21A2,MFNG,TMEM8A,PIM3,ARMCX3,CREB3L2,DESI1,INPP4A,TRAIP,SLC35E1,LAS1L,MTA1,OSBPL10,STK36,HKDC1,CKS1B,DPH2,NOM1,RASSF4,PKD1,ACADM,ARF1,NFKBIL1,CNN3,ALPP,DNAL4,TBC1D10B,MFSD4A,UBR4,LAMB1,SPINK5,SACS,CLIC5,AP5Z1,FANCC,ITGB3BP,PQLC1,BCR,SETD5,TM9SF4,KREMEN1,SLC38A5,ZC3H11A,CCM2,PDZD4,KDM4A,XKR8,DTNB,PABPC1L,TP53BP2,EWSR1,RGR,COPS6,PDLIM4,STIMATE,NPDC1,CCZ1B,NEBL,TSPAN32,GSDMB,ANKS6,ALB,LBR,ISM2,AC114490.2,ARIH2,RAB11FIP3,PSD,MMP11,TMCO4,MZT2A,CREM,IARS2,UBAC1,CACUL1,ATRAID,TOP2B,C3orf58,ZNF789,AL080251.1,SLC4A2,EIF3B,JAK1,MFSD2B,SH3BP1,AMT,PCMT1,LY6G6C,KDM4C,LAMC1,NRXN2,STX1A,LZTR1,MAST2,TNIP2,DGKQ,MARCH6,ABCC3,NR1I3,ATP13A2,C5orf34,ZDHHC11,INPP4B,PXYLP1,TLR1,SIL1,THAP9,SLC9B2,CHIT1,AMN1,ARL10,RGS7BP,IRF2,IFT122,SDHA,HK3,TRIP13,SPAG9,ZNF451,NADK2,SH3BP2,CEP72,GYPB,WDR36,PCDH1,ZFAND1,TBC1D9B,ATP6V1C1,PIEZO1,RRNAD1,NSMAF,TRIQK,TSPAN18,STC2,ENTPD4,MROH5,ATP6V1H,MTSS1,PRSS55,ERICH1,PHYHIP,REEP4,AARD,DIS3L,ABCA7,PPP6R3,KCNK7,USP47,NUP214,RPS3,C11orf16,ME3,ST5,PACS1,TK2,PRDM10,RIC8A,ARMS2,SULF1,ANO

	1,TPP1,CDKL1,CTBP2,INTS11,TP53I11,PCNX3,IGLL5,MOB2,MAJIN,CAMSAP2,RCE1,ACAT1,PPP1R16A,TKFC,POLR2L,NUMA1,PTPRO,PLEKHB1,ARAP1,ACSF3,RELT,PDE1B,NCAPD2,IGHMBP2,SLC22A1,FAAP100,MMP20,M6PR,WDR74,DNAH10,MGP,SLC39A3,SUOX,DDI2,TPH2,HECTD4,HVCN1,KRT78,NTN4,GAS2L3,PFKM,CSAD,DUSP6,PLXNC1,DAZAP2,KRT6A,CA6,DDX23,TSPAN8,FICD,ESPL1,HECTD1,ACTN1,PSEN1,ASB2,PCNX1,MIPOL1,SIPA1L1,PPP1R13B,NIPA1,IDH3A,GATM,MAN2A2,EIF5,RNF31,GABPB1,ADCY4,TLN2,GCNT3,DNAJC17,ALDH1A2,PBX4,GMPR2,SEMA4B,CEP152,FAH,SLC9A5,ATXN2L,PLA2G4F,PRMT7,GALNS,CASKIN1,AC090360.1,EXOC3L1,AMFR,CLCN7,CCDC33,ARMC5,C16orf58,NOL3,CNOT1,TAF1C,ZNF688,VAC14,BAIAP3,DDX19B,NLRP1,NEIL1,ABCC11,GEMIN4,SLC25A11,PELP1,CIC,SPACA6,FN3KRP,TSR1,CLUH,FAM57A,CHRNB1,SGSM2,STRA6,CORO7,AKAP1,HCFC1R1,WNT9B,URI1,NAA60,RASSF5,DUS1L,SMARCD2,TTC39C,MYO15B,ADCYAP1,NARF,TMEM94,ITGB4,RAB31,GDPD1,LRRC45,OGFOD3,CDC6,CCDC57,FLOT2,GHDC,WDR7,TEN1,MAU2,HAUS5,ZNF793,MAP3K14,RBFA,HIGD1B,CXXC1,GPATCH8,FHOD3,ROGDI,TMEM161A,ATP9B,EFTUD2,ACTN4,ZNF532,EIF3G,ALPK2,ZACN,LIG3,EPS8L1,NAGLU,SLC7A10,JSRP1,CTAG1A,ARHGEF1,ADGRE2,ECH1,CD22,ILVBL,MCOLN1,HPN,CCDC97,ZNF17,F2RL3,TMEM38A,PPFIA4,EMG1,KCNN4,LIPE,VAV1,FSD1,HNRNPL,SIGLEC7,ZCCHC18,LCE1C,ARFRP1,GRM4,AL034430.1,MYO19,UNC93B1,PCGF2,MED26,TTLL13P,SPPL2B,AC008162.2,SIK1B
**hsa-miR-103a-3p**	CAMKK1,CNFN,CD69,MXI1,APC,GNB5,TCIRG1,EXPH5,KCTD7,SEC16A,KCNMA1,PKNOX1,TNFSF12,FASTK,HRASLS5,MAP2K1,FAM53A,NOSTRIN,GPR137C,ZAR1,APP,ZFYVE26,MYO9A,SMOC1,SOX13,CFAP46,TTF2,ZNFX1,GLO1,MAP3K8,REL,CYP1A1,TSPAN4,GATD1,NONO,KMT2B,IFT22,MFSD13A,MED20,DGCR6,ZNF879,AL139260.3,DLG2,TMEM72,BRDT,STAT3,PPP1R2,LAMB2,TMEM54,SNX17,MFN1,SLC11A1,ASB18,HECW1,CERCAM,SMARCD3,NDUFV2,DENND2A,TOGARAM2,PLIN2,MMACHC,MRPL38,RNF146,SPTLC1,GTF2IRD2,MPP3,CAPN10,ITGB1BP2,CFP,PTP4A2,TTLL3,SLC2A11,FAM45A,SUB1,MGAT1,ZNF346,CNGA1,FER,ELMOD2,CORIN,WDR17,MZB1,ARL15,UBE2Z,ZNF706,ITK,ACAT1,DDX25,CAPN5,GDPD5,DEAF1,TRMT9B,MRPL16,FKBP4,MLXIP,ARAP1,SLC38A10,LETMD1,ACTR6,ATAD2,DAAM1,TMCO5A,DET1,IVD,RPS27L,KATNBL1,MAN2A2,SQOR,METTL22,STX1B,GINS3,SPG7,CDH3,WDR59,PPP1R27,MPDU1,MYCBPAP,ZZEF1,CCNE1,TMEM105,LASP1,CCDC57,ERBB2,CLPTM1,FOSB,KDSR,GPI,DNAJC7,KEAP1,KDM4B,ZFR2,CACNA1A,DAPK3,SIPA1L3,LIG1,ZNF17,IRF3,SNRPA,SELENOW,USP29,EMC8,ELAC2,MMRN2
**hsa-miR-122-5p**	PPP2R5B,GATC,VPS53,SLC25A34,XPO6,SWI5,NUDT1,GPRC5C,OR2AK2,BGLAP,TSPAN2,BPIFB3,CFAP74,RAB3IP,CAP2,DENND1A,DOT1L,FAM118A,HECW2,G6PC2,VTCN1,FHIT,SLC11A1,THADA,RAC1,CMTR1,STX7,ZP3,SFXN3,PPP2R2D,INPP5K,ZAP70,DMXL1,ETF1,SLC2A9,FUT10,GEMIN5,PLEKHA7,C11orf49,MROH1,FPGT-TNNI3K,GNS,APPL2,UBAP1L,FANCA,BEAN1,SIRT7,RAB8A,DNMT1,CCDC155,NUMBL,TMEM185A
**hsa-miR-125b-2-3p**	GUCA1A,PTBP3,ISL1,NPC2,SLC52A1,EDAR,HINT2,MUL1,GCKR,SPIB,AWAT2,RPP30,PLEKHG4B,BTG2,SOX8,KCNT2,CAPN13,PF4,CCDC174,B4GALT2,CCPG1,BANF1,TPRX1,WDR45,RPAIN,HCAR2,BRCC3,RTN4RL2,AGAP3,SH2D6,ZKSCAN7,NPAS3,CHRD,THSD4,TBX19,NR1I3,SARDH,MMRN2,KLF17,CLCN5,CYP1A1,CIITA,CHD2,DOK5,NRL,IL25,ARID1B,C19orf24,CD8A,VDAC2,CFAP65,TMEM250,IL1R1,AC03745

	9.1,DPCD,PRSS45,WDCP,AC023509.3,TPR,ZNF674,GLP2R,KCP,ST3GAL5,MICAL3,MRPS31,TRANK1,LAMA5,ABCA2,TFIP11,GTF2I,C2orf42,LETM1,NOL8,PCOLCE,DLG5,ACSS2,ECE1,RNF182,CERCAM,VTI1A,NIT1,ECE2,KRBOX4,SKIL,PPP2R1A,IL11RA,C21orf2,C2orf70,ECHDC2,SPINK5,ROR1,DNER,HLADOA,LRP1B,TMLHE,RGS3,TAB1,IL9R,FAAH,CCT3,ZNF491,LY75,CENPL,PCOLCE2,PER3,MIA3,DGCR8,SSR3,CNIH3,AURKAIP1,GLB1,RPL9,ACAD9,APBB3,TSC22D3,RCHY1,FAM81B,CANX,RNF175,POLR2B,PALLD,CNOT8,CARD8,SPIDR,ZFHX4,ZNF706,ZNF395,MYOZ3,DECR1,CEP57L1,THAP8,IFITM1,PLEKHA7,MTA2,ZNF692,LRP5,HCAR3,C11orf88,SLC37A4,REPS1,SMPD1,C12orf60,NPEPPS,CRACR2B,ATG16L2,ACACB,TM7SF3,C2CD5,MED21,TARSL2,CCDC91,MPHOSPH9,CD163L1,IBA57,CAPS2,ACVRL1,TWF1,MED13L,MAPKAPK5,TNS2,HDAC4,KRT86,LIN52,NAB2,EAPP,PAK6,TIPIN,CDH11,CARHSP1,UBR1,NRG4,AC026464.3,MYO9A,EARS2,IL4R,ATXN2L,FAN1,CPEB1,ANKFY1,P2RX1,TMEM100,RPL23,PPIAL4A,ASPSCR1,PPIAL4F,PPIAL4E,AC243756.1,PPIAL4C,ZNF559,MOB3A,NKIRAS2,TNNI3,HDHD2,GLYR1,EFTUD2,C18orf25,LIN37,KLK2,LTBP4,ZNF749,PEG3,PHLDB3,ZNF772,AC012254.2,DUSP22
**hsa-miR-125b-5p**	CYP26B1,MLF2,UBOX5,BCKDK,ANAPC15,PXN,DOK1,ARHGAP40,PACSIN1,LRFN3,MAP3K10,USP29,KMT5C,DDB2,TIMM10,AKAP2,HHAT,OSBPL5,MUL1,ANK2,SFRP5,GFOD2,LRRC46,ZNF593,PTGES2,FAM160B2,CRYAA,FAM207A,IKZF3,MAPKAPK2,ANKRA2,ANPEP,TSC22D4,VPS37C,UTF1,MAP3K11,ABLIM3,MATK,SPEG,BTBD9,CDPF1,AMBRA1,ASB2,CSH1,TMEM107,GADD45GIP1,EIF4E1B,TMEM184A,EHD1,ZNF7,LMNB2,IFNLR1,CTAG1B,HDDC3,SNAI3,SIGIRR,ZNF555,TMLHE,SELENOV,GGT7,OPRL1,ZNF385A,CRTC1,SECISBP2,SP100,SLC26A9,HIST1H2AD,VCX,WDR31,COL11A2,COL18A1,NUTM2B,EPB41,LRRC69,PSMD13,NUTM2G,NEIL1,ADAM11,SERPINH1,EPHA2,ZNF707,SSU72,SIRT5,NT5C1B,BAK1,R3HDM4,SMG5,S100A3,CTAG2,STMN3,DNLZ,AIF1L,GDAP1L1,MANBAL,HSPG2,PLA2G5,SKIV2L,PROSER2,NUTM2D,NUTM2A,RPUSD3,WIZ,PTOV1,CSH2,MED20,UBE2D3,ANKRD13B,CENPO,EMP1,CCDC125,TAGLN2,OTOG,HMX1,CCDC134,UBE2I,COMT,HIC2,TTC7A,VWA3B,MAGEA2B,AFF3,RBM44,MTHFR,DENND6B,MUM1,MECP2,RPS28,SLC19A1,ANLN,SERPINE3,ZNF839,RFC5,POR,CTU1,ADORA2A,C20orf173,DBNL,NUTM2E,AKR1D1,ENTPD6,ZNF780B,HLAF,TOP3B,RGSL1,CRP,EXO1,PNCK,RARRES3,ENO2,FZR1,SPAAR,OSBP2,TMEM131L,MED22,NEK6,AC037459.1,SMARCD2,ADAM12,FANCG,ASCC2,OGFR,C8orf58,SEMA4F,ANKRD60,LYRM4,CFAP65,TBC1D2B,HM13,MGST3,CPT1B,UROD,PDGFRA,DUSP18,PRR14L,SGSM3,C1orf61,RNF103,MIB2,SEMA4D,WDR27,CLSTN1,MPZL1,ACAA1,COL11A1,MOV10,GP6,SSR2,COL21A1,ST7L,SFI1,FGFR2,PARVG,EXOC4,TMPRSS2,NOC2L,TTN,SLC4A11,TSC2,PTPRE,TEP1,PARD3B,ARPC1A,P4HTM,TM2D1,DNAJC11,AUP1,STK11IP,DHX30,TLE6,SNTG2,PARP3,CEP250,LCK,SLCO2A1,ARR3,ARHGEF3,MFAP2,SYNGAP1,PLCD1,NEU1,FES,SEPT8,ABLIM1,QARS,EPS8L3,GPRC5C,ZAP70,PCNX2,NDUFA3,ACP1,LRRC29,RHBDF1,PTPN14,TBC1D5,MUSTN1,PHF1,NR1H3,DNAH1,TCTA,HES2,INTS11,C7orf50,PCYT1A,PIGL,ZBTB48,MDC1,CNBD2,AURKAIP1,ASB3,PTPN18,REG3A,CYB561D2,IKBKG,AGRN,PTK7,NCOR2,MAGEA8,PSAP,CLCNKA,TPI1,ACTR8,HDAC4,SLC2A4RG,HHATL,DNAH10,ADAM10,REG3G,HDAC11,ZC3H12D,CEBPA,P2RX4,FLT4,F12,CDK10,WDR41,PRELID2,COL4A5,PIGG,LYRM9,PDK2,FRMD1,IDUA,ALG1L2,SLC52A1,HARS2,CHCHD6,TRIO,RMDN1,STMN4,GLI4,LYPLA1,ADAM18,AP3M2,DECR1,RAB11FIP1,PNMA2,RPS6KA1,SIGLEC10,FHAD1,MCAM,TP53I11,SPAG11A,GSDMD,PTPRU,TMPRSS13,TTC12,PRMT1,ZNF250,CHID1,ZDHHC5,SPAG11B,GATD1,BRMS1,ST14,DPF2,NDUFS8,SIDT2,E2F8,GRK2,ST5,PLEKHA7,GRAMD1B,SART1,DPP3,OLFML1,TMEM258,TMEM134,MPHOSPH9,CD6,SLC6A12,SFSWAP,VWF,VTN,HIP1R,MMAB,PGA4,PGA5,C1RL,MACROD1,ST8SIA1,PAH,PAX8,IFI27L1,SLC25A29,I

	TGA7,IDH3A,CCDC33,CD79B,ZNF710,DPP8,PHGR1,CMIP,RSRP1,PRSS36,SPIRE2,CASKIN1,MVP,CDH11,KDM8,RBL2,RPAP1,KIF22,EME2,VAC14,CLEC18A,BRICD5,KATNB1,PLEKHG4,ABCC11,IL34,SLC25A10,CNTROB,SPACA6,AC139530.2,ITGAE,DVL2,CHRNB1,SGSM2,TXNDC17,MRM3,NPLOC4,SLC46A1,ARHGAP44,MAP3K3,RBFOX3,MARCH10,ICAM2,RAB11FIP4,CENPV,RPL36,TBC1D29,TNS4,SCN4A,RAC3,VAPA,GHDC,CATSPERG,SPPL2B,STK11,TMEM161A,DOCK6,CARM1,CXXC1,C19orf66,MAP4K1,PRKAR1A,PODNL1,VMP1,CIRBP,ADAMTSL5,POLRMT,FIZ1,ZNF234,ACP5,APOC4,EPS8L1,C19orf12,COQ8B,PET100,C19orf44,AXL,KLK8,GPR108,FCHO1,ZNF414,SPTBN4,ANGPTL4,SNRPA,SIGLEC7,KXD1,NMRK2,KHSRP,CTAG1A,CD37,SLC25A42,ZNF329,NAPA,TP73,AC116565.1,SSTR3,YTHDF3,HTR2A
**hsa-miR-1271-5p**	ADCK2,GNA11,HDAC7,AQP2,CBX6,TRIB3,ZMYND10,IL17C,PDCD2L,SDF2L1,EEFSEC,ESPL1,BCO1,FAF2,GIPR,DBH,BUD23,ERBB3,GPR17,AIG1,EML3,SLC24A2,TYSND1,GOLGA6A,FASTK,C9orf24,PACSIN3,PLK1,NLRP4,C2orf54,ASPHD1,GPR137,CLASP2,TEX44,FUT7,CABP1,ZNF771,RPS15A,NLRC3,OR8B8,MCF2L2,MT1E,ID2,FAM129C,BET1L,MT1B,DOK7,CDK11B,ING4,RPS21,SDSL,ATG16L1,MAGED2,GIT2,ENTPD6,MYH7,CDK11A,ITIH2,GOLGA8A,CDC42SE2,ODF2,SLC29A3,SKIV2L,MGME1,PRDM16,AC026461.4,CPXM1,GREB1,GPRC5C,BBS1,CELF6,MIEF2,CEP250,RGPD2,SAMD12,SPATA13,CRIP1,RGPD1,THRB,CARF,IFI35,MX2,C21orf33,TVP23C,PROC,POLR1C,SHANK2,SPHK2,EFHD1,OXSM,IFRD1,CARD10,MBD3,C11orf68,CBX5,DBNL,HNF4A,FAM160B2,PLA2G6,CABLES2,F8,FAM171A1,GRAMD4,COL21A1,MBOAT2,SLC12A8,GRAMD1C,TSTD1,CHRNA4,MT1HL1,MIB2,FDPS,WDR31,RAPGEF4,ANO10,ADORA2A,PLXNA3,THADA,CRELD1,IL17D,KIAA2012,HPCA,PRKCZ,LAMB1,SACM1L,P2RY11,RIPOR2,DENND2D,ABCF2,RNF220,RAB34,ERGIC3,ASAP3,RUNDC3B,UTP6,PRPSAP2,GUSB,USP19,EPHA2,TIE1,LAMB2,GBP5,STARD3,AGPAT3,GKN2,MGMT,TBRG4,CUL3,MEGF11,EVA1C,CSTL1,KCNIP3,SREBF1,IFRD2,DRG1,ATP13A1,TTC22,CSF3R,DRC3,SHISA5,ANKRD16,TPRG1,SRC,HDAC6,DMAP1,AZIN2,PLP1,LRRC2,LAMA5,C1orf109,MBP,IQGAP2,PCDH1,KIF13A,ANKHD1,PPAT,HSD17B11,SPATA20,NGFR,MXD3,RNF44,QRFPR,FAM200B,METAP1,ARAP3,PAPSS1,ANXA6,OSR2,GRIA1,FAM83A,SLC39A14,TAF2,ENO3,ARFGAP2,GOLGA8G,CTTN,TMEM262,PIDD1,TPP1,GOLGA8F,PPP1R16A,MMP7,IMMP1L,CDKL1,CREB3L1,PEX5,IPO8,CARS2,NLRC5,CCND1,PRH1,LHX6,SPECC1L,MAP1S,ANO2,OLR1,PACS2,TPCN1,GNPTAB,AL928654.3,CNR1,PRKCH,PTGDR,TTLL5,SLC35F4,MTHFD1,ALDH6A1,TVP23CCDRT4,GLDN,SLC12A1,TM2D3,IGDCC3,NIPA1,MTFMT,SQOR,FAM214A,MT3,GOLGA8S,ATXN2L,GOLGA8M,GRAMD2A,AC090527.2,MAP1LC3B,ADCY7,GOLGA8H,GTF3C1,GGA2,GOLGA8J,GOLGA8T,DDX19B,MAN2C1,VPS53,TVP23B,SLC43A2,SIRT7,RNF213,CFAP52,CYB561,TMEM241,TOP2A,ARHGAP44,MKS1,SAP30BP,WIPF2,BAHCC1,LSM7,AES,ARHGAP33,GIPC1,G6PC,USP32,CDC37,MBD1,CIRBP,AC092073.1,GHDC,GTPBP3,ZNF665,TIMM50,HNRNPM,F2RL3,DLL3,KLK2,PNPLA6,DUSP22,CCL15CCL14,NTMT1,MLLT6,SRCIN1,CCL23,CCL15,PTPRQ
**hsa-miR-130b-3p**	PRTN3,BCL2L14,TCEA2,PPA2,MRPL37,CNIH4,STXBP5,IPPK,OR10A4,ATP6V0A1,OR10W1,THOP1,AGAP1,WDR43,TRIM2,ADGRG3,AOX1,ANKS6,CXorf65,DLG5,ACSL6,USP37,EXOSC7,ATG2B,PRKD1,USP34,ABL2,LYRM2,FNTA,MTMR9,MYRF,FKBP4,RAB5C,KCNH3,RPS29,APH1B,CNOT1,AKT2,AC099811.2,NDUFA7,C3,INSR,TIMM50,AC010323.1,MFSD14C
**hsa-miR-133a-3p**	ITGA3,B4GALT7,NGFR,TRO,TRAM2,CDC34,MORC2,NME4,POP4,FBL,SIPA1L3,AEBP1,CDX1,INO80B,DLGAP3,PDZD11,KLRK1,MRPS2,SEC61A1,PYCARD,PIN1,FXR2,CNN1,PRDM12,YIPF2,NAPSA,PPP1R1B,FADS2,TTBK1,CACNG4,GGT5,PPP1R12C,KCNT1,COL5A3,CHFR,SCAMP2,ARHGDIA,SLC47A1,SEC22C,ADAMTSL1,SOGA1,SPARCL1,OSCAR,OBSL1,PDXK,NAGS,FCGR3B,TRIM17,COL6A3,CLEC3B,SENP2,C

LEC1B,PRRT2,SLC50A1,KCTD19,DDIT4,SSH3,CLDN15,DNAI2,GABRB3,MMP14,RP
L7A,STOML1,RORC,MARCH8,SF3B2,PDZD3,FOXJ1,FOXL2,FAM187A,CMTM1,NEL
FA,SERPINA11,NCKAP5L,MMP25,MXD4,ZNF385A,FBXL19,BRAT1,TRIP12,TRADD,
DHX30,NPHS1,IL27,FGF17,LAT2,PIK3CD,SREBF2,PNMA5,MRPL55,ENAH,FCGR3A
,PFDN2,DNASE1L1,EDN3,SLC2A6,PREX1,LRRC73,FAM167B,KIF12,MSANTD3,MG
AT5B,STK19,RSG1,MPIG6B,DLG2,ACTN1,CNNM4,PLXDC2,ACSF3,SHROOM1,NU
DT2,CDKN2A,FUS,C1QTNF7,LNP1,IL17RE,ABL1,PTTG1,KCNK7,CELF6,FGFR1,GST
P1,COL13A1,MYL5,ARVCF,DPP6,ABLIM2,CCDC103,SLC35G6,ATP5MFPTCD1,PSM
B9,C2orf76,TRAF2,DDAH2,GRPEL2,RASAL1,EPCAM,SFXN4,CD22,CERCAM,BRPF1
,OGFOD2,CEP250,TTC38,FAM182B,GIGYF2,SDCBP,PRDM1,PICK1,MRPS18A,RPL10
,MGAT1,TULP1,LSP1,KCTD15,PIGV,RLN3,MICAL1,PILRA,MLPH,SERPINE2,POLD
2,NCOA6,SH3BP2,MMP23B,EMID1,SEC14L2,GCK,PCED1A,FAM20B,TSSC4,LCP1,E
LF3,CCT3,ZNF185,PILRB,TTC31,ASCC2,RASA4,ACAP2,SMIM34A,FCAMR,DMKN,
ACTR1B,RHBG,GSS,COL9A3,TGM2,PLEKHA3,BACH2,KLC4,ARFGAP3,PLA2G6,TT
LL6,CLCN2,PFKL,DXO,ZNF862,STK40,KEL,NADK,MAMDC2,SCAMP4,SHANK1,A
PP,MRPS5,ABCA2,PEX5L,NDUFB8,TFDP2,CCDC13,LANCL2,RHBDD2,RNF13,PSO
RS1C1,MSH5,SNORC,CYP2J2,SURF6,B9D1,FAAH,CRAMP1,SH2D3C,WDR54,CXorf5
6,HSPH1,EFNA3,MYO1A,KLHDC8B,IL1RN,ACAP3,TBC1D2B,MXRA8,AL096711.2,S
SBP1,RRAGB,SPAG8,CPA1,FAM129B,HMGCS2,NRP2,DNASE1L3,PDZRN3,NFKBIE,
CACNA2D3,MAGED4,BCAR3,WHRN,MAGED4B,EXOSC7,IZUMO4,ZNF783,NT5C3
B,CNTN2,AC093668.2,YPEL5,MLF1,ZNF391,PDS5B,EZH2,PKN3,POLR3H,TAF1,CO
G5,OPHN1,CLASP1,MYCBPAP,ZNHIT1,PPIL2,PRSS53,PRKCB,CDS2,TRIM56,FUBP
3,CCDC150,NISCH,NGEF,DDC,PRRC2B,PNPLA7,RREB1,CBS,DOPEY2,ARPC1B,NC
OR2,RABL2A,NICN1,DEPDC5,TSC22D1,LRP8,OGDHL,GYG1,UBE2G2,HM13,ARH
GAP8,CEBPA,SH3RF1,PGM3,MZB1,TRIML2,PITX1,NFKB1,KLHL3,HK3,ACAD9,SE
C24D,FAM193B,IPO11,FGFRL1,SLC10A7,SRPK1,MGAT4D,EXOC3,AXDND1,CFI,CO
RIN,COL12A1,ANKHD1,ATP8A1,NR1I3,ABHD18,BFSP2,LMAN2,CPLX2,ACOX3,Z
NF704,NDRG1,VIRMA,TMPRSS4,AC105052.1,SNX31,GRIA1,MGAT4B,SORBS3,VDA
C3,SLC39A14,ST3GAL1,TRIQK,NKX63,TBC1D9B,PITPNM1,CCDC17,C1QTNF5,ZNF
250,TESPA1,ZNF195,PPP2R5B,PPP1R16A,NKAPD1,CHMP4A,TYK2,BACE1,ARHGE
F4,ECE1,ZNHIT2,LRP5,AMPD2,TMEM86A,SART1,CKLFCMTM1,EFEMP2,MRPL17,
SMPD1,NDUFS8,BBOX1,DNHD1,STK33,PCSK7,ATM,SIPA1,P2RX3,TPCN2,GPR162,
KIAA1551,PDE2A,RHOF,CARS2,SRGAP1,SLC6A12,TRMT10B,EHD2,VWF,STAT6,ID
H2,NDUFA9,RASA4B,NOP2,MEIS1,KLRC4KLRK1,PLEKHB1,WSCD2,ZNF606,MAP
1LC3B2,MCRS1,MARCH9,EEA1,SOCS2,FKBP11,GATC,ARHGAP9,TNS2,ITGA5,ITG
A7,SDR39U1,SLC7A7,WDR25,CD63,AKT1,RPS6KL1,CRTC3,TPM1,GCNT3,RTF1,SLC
22A31,TK2,MYO7B,KCTD13,KLHDC4,CBFA2T3,NEIL1,CDH16,CEMP1,CMIP,ABCA
3,DHX38,NTHL1,LMF1,SMPD3,VPS35L,STARD13,SLC38A8,LDHD,TCF4,ADCY7,ZS
CAN29,MAPK7,AKAP10,PLD2,ATP2A3,ZP2,RPS15A,SLC26A11,AKAP1,PELP1,ALD
H3A1,DPH1,POLR2A,NXN,PER1,RAB11FIP4,ITGB4,LLGL2,PIEZO2,SUPT4H1,PROC
A1,SLC25A35,SEPT4,ARL5C,SLC16A3,EIF4A1,PRR29,ERBB2,PRKCA,TSEN54,CD300
LF,MTMR4,CDK12,RPL26,TMEM94,MUM1,PCSK4,UBXN6,MATK,TLE2,EDDM13,N
AT14,NEDD4L,PALM,BCAM,APLP1,POLRMT,ATP5F1D,AP1M1,RFX2,SPHK2,SH3
GL1,SNAPC2,NAPA,PGPEP1,ETHE1,MYDGF,IRF3,KCNN4,MRPL34,SLC17A7,ARH
GEF1,VAV1,PPFIA4,PPFIA3,MORN1,TBX18,CCL15-
CCL14,DGKK,AC239799.1,CTDP1

**hsa-miR-184**	ANKRD54,PEX11G,SF1,MTUS2,AMPD2,TMEM184A,NLGN2,ZBTB46,C20orf196,TCTN2,ATP13A2,CARM1,CACNA1G,TOMM20L,TRIM27,KLF6,TSSC4,PPP1R21,AURKA,MTA1,HMCN2,PTRH1,RLN3,MAP6,CLUH,METTL8,PABPC4,AXIN1,HM13,SLC26A9,PHF21B,TLE1,ABCA2,CDC25B,DPYSL4,FAM83F,S100PBP,SLC2A4RG,ST6GALNAC4,AKT2,TRDMT1,MTMR11,KRTDAP,TARS2,GAL3ST4,ARHGEF10L,CTSA,PHF24,CTSZ,ARHGEF7,DGCR8,TACC2,IFT22,PPP2R3B,LHFPL4,UGT2A1,CDK18,EXOC1L,EFNA5,SPAG9,MGAT4B,STK3,LRRC6,FUT2,STT3A,TMEM80,SIGIRR,UBQLN1,ROBO3,ZSCAN9,C1RL,ARHGEF40,NRDE2,TTC7B,PSMA4,PSME2,CACNA1H,ZKSCAN2,FAM192A,ABAT,RNF167,NXN,ELAC2,CTC1,GPRC5C,PTBP1,AP3D1,PIP5K1C,LTBP4,LGALS4,SIPA1L3,PAFAH1B3,IRGQ,KANTR
**hsa-miR-203a-3p**	SLC26A10,PRKG1,ACVR1,FTSJ3,FOXK2,CCM2,SIM2,SLC35B1,ITGA1,DDX55,GMFB,SUSD6,FUS,LSM7,APOC1
**hsa-miR-206**	GTPBP1,LY96,SOCS1,TCF7L2,TCTN3,NOTCH4,TRIM31,BSCL2,ELMOD3,ASCC2,TMEM178A,COL20A1,MRO,ZNF33A,SFTPC,FAM120A,AQP12B,AQP12A,ROBO2,PER3,GSTO2,SLC25A12,OCIAD1,RHOBTB3,ASPH,PDP1,GDPD5,ZC2HC1C,FBRSL1,TRAF3,CD276,FA2H,CTC1,ATP5F1A,SIPA1L3
**hsa-miR-335-5p**	SGK3
**hsa-miR-551a**	DVL2,RPL18A,PCDHB6,IFT43,PCDHB8,PCDHB10,NEUROG3,TYMP,TUBGCP2,SLC2A11,COX10,KAT6A,ADAMTSL1,FAM81A,FAM46B,ZFYVE28,ABCF3,GPR182,ANGPTL4,MMP14,TRPM7,C4orf50,TMEM150B,SORCS2,EWSR1,DYNC2H1,SNRPD2,TRPM3,VKORC1,AC008397.2,ANKRD35,ZNF707,C20orf203,CNIH4,RXFP4,CHTOP,CYB5R4,ZNF451,BTBD17,DCTN3,EIF1AX,GMDS,AKAP17A,MPP1,GPT,ATP2A1,SELENOS,ZSWIM8,ITSN1,CENPM,ELF1,PRSS40B,PPT2,POLM,PASK,VILL,RTL10,ANKRD62,CIZ1,CAP1,PBRM1,APOE,NR2E1,MAGEA10,LSG1,ZNF776,GTF2I,LCNL1,TNK2,ADAP1,SULF2,COLEC11,TADA3,GPAT2,SFTPA1,RGPD5,TMEM114,OGT,SNRNP27,MFSD13A,CAV1,AURKA,GNB1L,WDR74,PRODH,SEMA4F,DENND6B,EIF6,ECE1,DDX43,LTC4S,RND3,CLTA,RARRES2,PLCH1,RWDD2B,ZMIZ1,ACAP3,SCAF11,TFR2,MECOM,MATN1,PLCG1,RGS19,HES4,KIF6,HBA2,MX1,AKAP2,MCF2L,PANK4,TSC22D3,RCC1,SLC2A5,LRCH4,HBA1,IFT172,LZTS2,RGPD6,HLAC,KRBA1,RGPD8,SLC22A18,RMND5B,MEF2C,TMEM110MUSTN1,RFC1,DDR1,KIAA1191,BDH2,FAM149A,KCNQ4,CPE,SPON2,SPAG9,SERF1B,RPL26L1,NCOA2,ADAM28,MPP2,KIF13B,SLC39A14,RSF1,DLG2,BCLAF1,CRACR2B,TNKS1BP1,CHID1,AC135050.2,TRIM29,HINFP,EPS8L2,PITPNM2,OGFOD2,C12orf65,DDX11,UBC,VSIG10,P3H3,KDM2B,C12orf57,FAM180B,TIAM2,CRIP2,SPRYD3,KCNH3,NDRG2,KLC1,EAPP,SLC25A29,DGKA,ALDH1A3,ADCY4,PIF1,PHGR1,IGF1R,GLDN,BCKDK,NIP7,SPG7,GPRC5B,APRT,RPUSD1,ITGAL,CTU2,RIPOR1,FAM192A,GSPT1,SGSH,FLYWCH1,CTRL,FUK,CHRNB1,SLC47A1,FN3KRP,NAT9,AMZ2,PSMC5,SLC46A1,PSMD8,ADAMTSL5,CYTH1,SPRED3,CDIP1,ANKRD27,TBXA2R,GIPC1,PRKAR1A,TMEM161A,FAAP24,GADD45B,CPAMD8,ADAMTS10,NFKB2,KLK2,PPFIA4,SYT3,AC003006.1,VAV1,FAM156B,NDOR1,ANKRD20A3
