# Supplementary material for: A Comparative Analysis of Naïve Exosomes and Enhanced Exosomes with a Focus on the Treatment Potential in Ovarian Disorders
Source: J Pers Med. 2024 Apr 30;14(5):482. doi: 10.3390/jpm14050482 (PMC11122298; doi:10.3390/jpm14050482)
Supplement: Supplementary file 1 [file jpm-14-00482-s001.zip › supplementary data 2/Supplementary data, Table S5.pdf]

Name of molecule	Type of disease	Mechanism of Action	References
**Protein**			
EFEMP1	POI	Activating AKT signaling pathway	21, 22
HtrA1	Ovarian cancer	Enhancement of chemotherapy sensitivity	23, 24
PAM	POI	Direct role in response to estradiol and ovulation cycle	25
SDF4	Ovarian dysfunction	Improper protein folding	26
**miRNA**			
MiR-1-3p	Ovarian cancer	Inhibition of cell proliferation and metastasis in ovarian cancer through targeted gene suppression of c-Met and FZD7, Enhancing chemotherapy sensitivity	27, 28, 33
MiR-103a-3p	Ovarian cancer	Suppression of proliferation and angiogenesis via CHI3L1 inhibition	34
MiR-122-5p	Ovarian cancer	Suppression of ovarian cancer cell migration, invasion, and metastasis via targeting P4HA1 gene, and inhibition of CD147-mediated pathways	35, 40
MiR-1271-5p	Ovarian cancer	Targeting CCNG1 for ovarian cancer growth inhibition, Regulating mTOR signaling via E2F5, and deactivating Notch signaling through TIAM1 targeting	41, 42
miR-133a-3p	Ovarian cancer	Ovarian cancer development inhibition via Wnt/β-catenin signaling pathway suppression	43
MiR-184	Ovarian cancer	Induction of apoptosis and inhibition of cell proliferation	47, 48
miR-203a-3p	Ovarian cancer	Regulation of ovarian cancer proliferation via Akt/GSK-3β/Snail signaling pathway and targeting CXCL1-mediated effects on PI3K/Akt, PLC/PKC, Ras/Erk, and JAK2/STAT3 signaling pathways	49, 50, 51
MiR-206	Ovarian cancer	Targeting c-Met to suppress AKT/mTOR signaling pathway activation, Inhibiting cancer cell proliferation, migration, invasion, and inducing apoptosis, alongside KIF2A targeting, Targeting CCND1 and CCND2 to inhibit	52, 53, 54, 55

		proliferation, progression, migration, and invasion of ovarian cancer cells, Targeting PFKFB3 to regulate cell proliferation via GLUT1, PFKFB3, and FAK, Targeting CDK4 to increase chemotherapy sensitivity	
miR-125b-5p	PCOS	Targeting Pak3 to activate ERK1/2: Inducing estradiol production and inhibiting granulosa cell death and testosterone production	59
miR-130b-3p	PCOS and POI	Targeting PCOS-related genes DENND1, ZNF217, RAB5B, LHCGR, ERBB3, and KCNA, Interact with insulin signaling via the MAP kinase pathway, targeting ZNF217 for modulating androgen secretion, Targeting PTEN to modulate the PI3K/AKT/mTOR signaling pathway, Resulting in suppression of apoptosis and promotion of cell survival, Impact on SMAD, crucial for granulosa cell proliferation	60, 61, 62, 63, 65
