# Supplementary figures and images for: A Comparative Analysis of Naïve Exosomes and Enhanced Exosomes with a Focus on the Treatment Potential in Ovarian Disorders
Source: J Pers Med. 2024 Apr 30;14(5):482. doi: 10.3390/jpm14050482 (PMC11122298; doi:10.3390/jpm14050482)

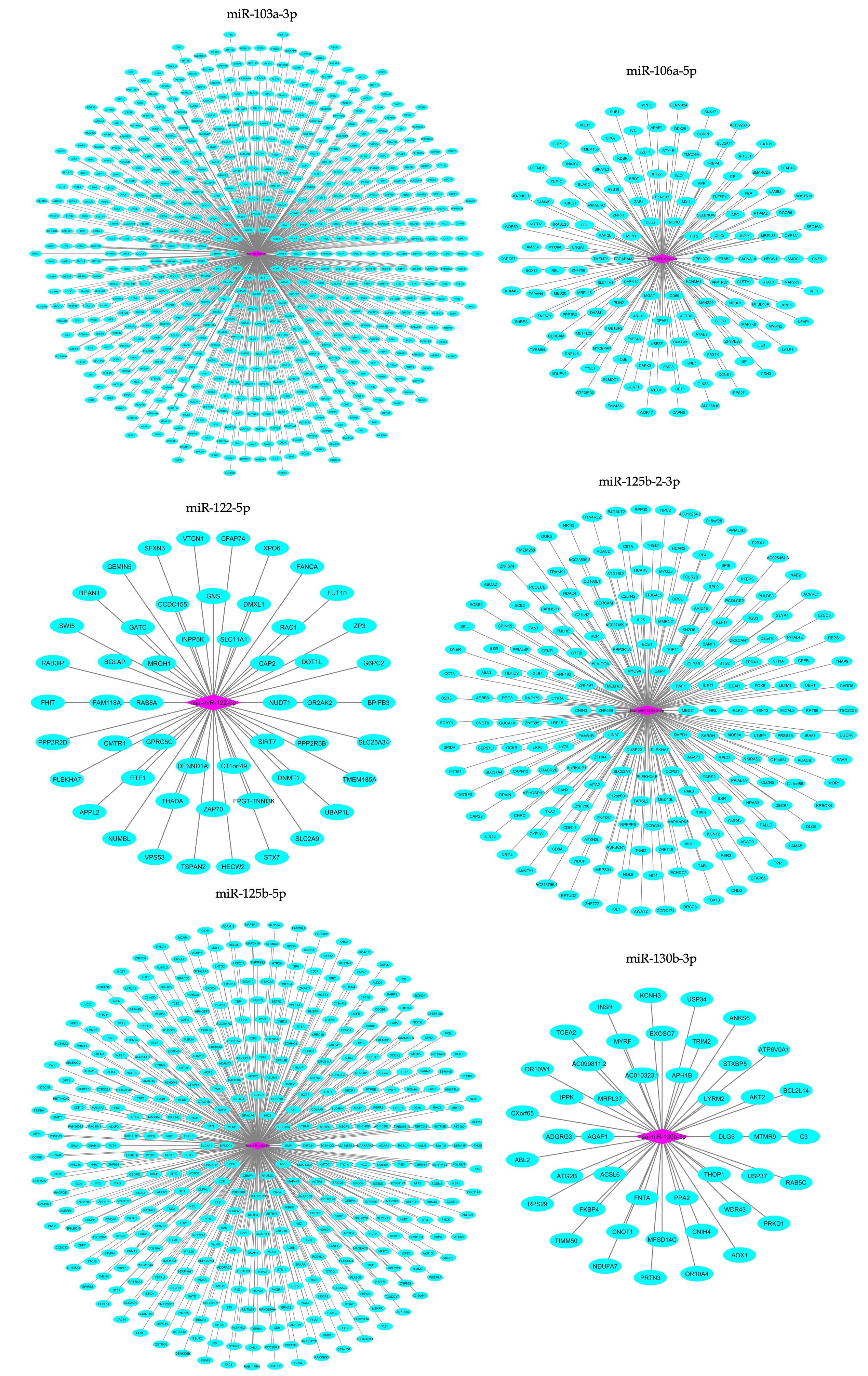

Supplement: Supplementary file 1 [file jpm-14-00482-s001.zip › supplementary data 2/Supplementary data, Figure S1-part1.jpg]

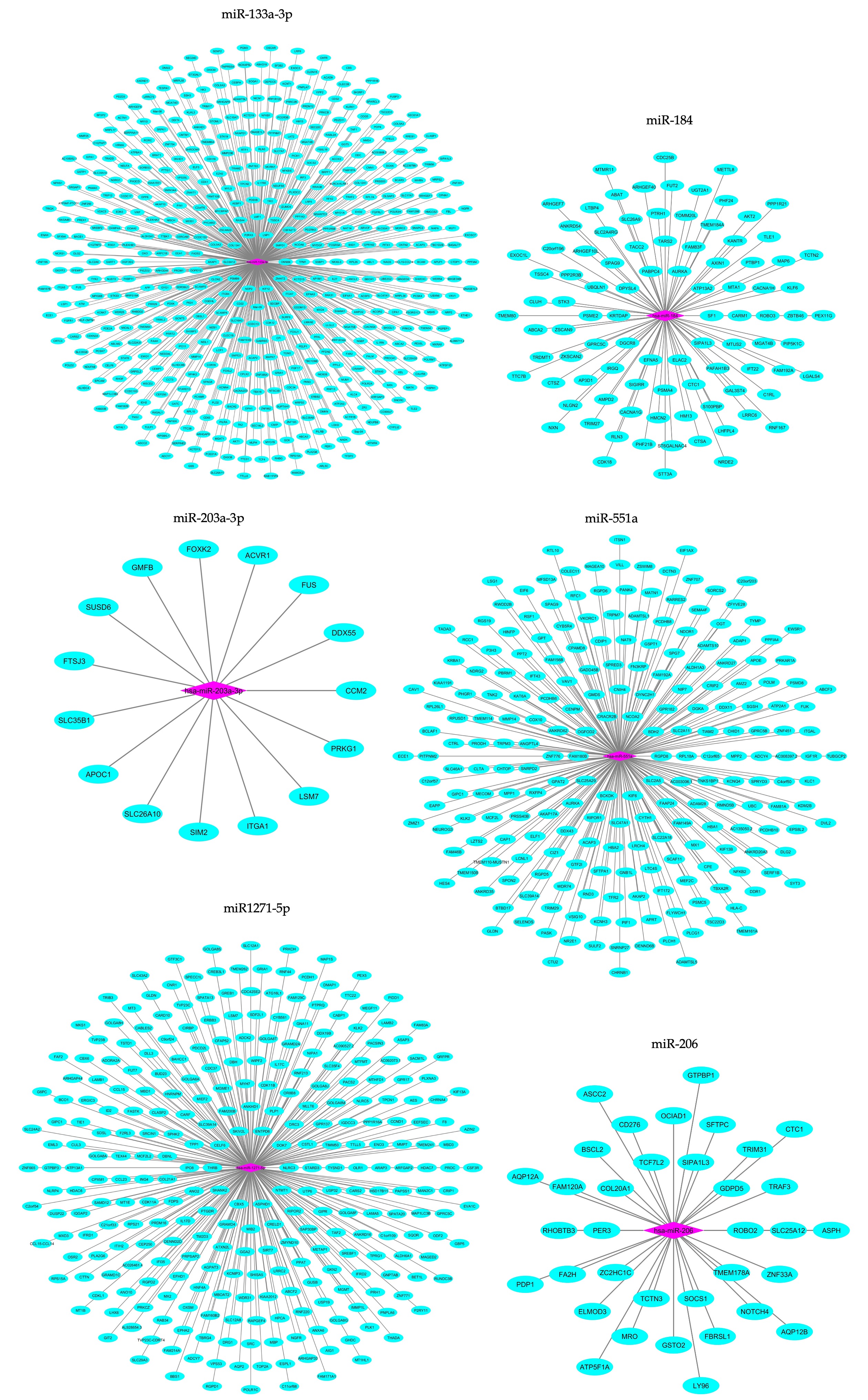

Supplement: Supplementary file 1 [file jpm-14-00482-s001.zip › supplementary data 2/Supplementary data, Figure S1-part2.jpg]
